# Two *Eremogone* (Caryophyllaceae) genomes provide insights into alpine adaptation

**DOI:** 10.1016/j.pld.2026.05.008

**Published:** 2026-05-21

**Authors:** Lizuiyue Li, Nannan Zhang, Liqiang Hou, Xiaozhu Guo, Huizhe Feng, Yongzhi Yang, Bin Tian

**Affiliations:** a Yunnan Key Laboratory of Plateau Wetland Conservation, Restoration and Ecological Services, Southwest Forestry University, Kunming 650224, China; b Shangri-La Potatso National Park Bita Lake Plateau Wetland Ecosystem Observation and Research Station of Yunnan Province, Kunming 650233, China; c State Key Laboratory of Herbage Improvement and Grassland Agro-Ecosystems, College of Ecology, Lanzhou University, Lanzhou 730000, China; d College of Life Sciences, Zaozhuang University, Zaozhuang 277160, China

**Keywords:** Caryophyllaceae, *Eremogone*, Alpine adaptation, Whole-genome duplication, Comparative genomics, Transcriptomics

## Abstract

• Chromosome-scale genomes of two *Eremogone* species.
• A recent shared genome triplication shaped Caryophyllaceae evolution.
• Reconstruction of the ancestral Caryophyllaceae karyotype (ACK, n = 9).
• Biased gene retention after triplication is linked to alpine adaptation.
• Multi-tissue transcriptomes link regulatory divergence to alpine adaptation.

Alpine ecosystems represent some of the most extreme terrestrial habitats for plant life, characterized by low temperatures, intense ultraviolet (UV) radiation, and limited oxygen and soil nutrients (Körner, 2021; Zhang et al., 2026). To survive these harsh conditions, alpine plants have evolved diverse morphological, physiological, and life-history adaptations. Common morphological traits include dwarfism, cushion habits, and reduced leaf area, which often arise through convergent evolution (Körner, 2021). Beyond these physical changes, alpine adaptation is driven by a complex suite of genomic innovations, such as single-nucleotide variations, structural variations, transposable element dynamics, and polyploidy (Guo et al., 2018; Chen et al., 2019; Zhang et al., 2019; Li et al., 2023). Through these genomic changing, alpine species have significantly enhanced their capacity for UV protection, DNA repair, cold acclimation, oxidative stress tolerance, and, in certain systems, hypoxia response (Li et al., 2023; Zhang et al., 2024).

Among these factors, polyploidy and post-polyploid evolution are recognized as primary drivers of adaptation to extreme environments (Adams and Wendel, 2005). Polyploidization rapidly generates genetic redundancy and dosage shifts, providing the raw material for neofunctionalization, subfunctionalization, and regulatory rewiring to enhance environmental tolerance (Soltis and Soltis, 2016). However, the long-term genomic stability and functional differentiation of polyploids are largely shaped by subsequent diploidization (or rediploidization). During this process, genomes undergo extensive gene fractionation and chromosomal rearrangements (Schnable et al., 2011; Wendel et al., 2016). Notably, this evolutionary process is non-random, where retained duplicates undergo changes in sequence and expression that facilitate adaptation to changing environments (Mandáková and Lysak, 2018; Wang et al., 2025). More broadly, duplicated genes arising from different mechanisms, such as WGD versus small-scale duplications, often differ in their abundance, selection pressures, and evolutionary trajectories (Conant and Wolfe, 2008; Mandáková and Lysak, 2018; Wang et al., 2025). This underscores the need to explicitly consider duplication modes and post-duplication regulatory changes when evaluating adaptation (Maere et al., 2005; Van de Peer et al., 2009; Qiao et al., 2019; Wang et al., 2025); however, how these patterns contribute to alpine plant adaptation remains largely unknown.

Recent phylogenomic analyses in Caryophyllaceae have revealed repeated polyploidization events associated with shifts toward colder climatic niches (Feng et al., 2024), raising the possibility that post-polyploid genome evolution contributed to alpine adaptation within this lineage. However, direct genomic evidence linking post-polyploid evolution to adaptive divergence between closely related alpine and lowland species remains scarce. Identifying the genomic basis of alpine adaptation through such species pairs is highly effective (Zhang et al., 2024), yet these comparative modules are rare. Here, we focused on the closely related *Eremogone* species pair: *Eremogone kansuensis*, an alpine cushion plant restricted to elevations above 4000 m on the Qinghai–Xizang Plateau, and *E*. *juncea* var*. juncea*, which occupies low-altitude habitats below approximately 2200 m. This species pair diverged recently (Yang et al., 2024), representing an ideal system for investigating genome evolution under the contrasting ecological conditions associated with alpine adaptation.

We generated chromosome-scale genome assemblies for *Eremogone kansuensis* and *E. juncea* var*. juncea* using PacBio HiFi long reads combined with Hi-C data (Fig. 1A and B). The final assemblies reached 1.82 Gb and 1.83 Gb in total length, respectively, and were anchored into 11 pseudochromosomes for each species (Figs. 1C, S1 and S2). Assembly continuity was high, with contig N50 values of 55.18 Mb in *E. kansuensis* and 9.81 Mb in *E. juncea* var*. juncea*, and scaffold N50 values of 165.42 Mb and 165.28 Mb, respectively (Table S1). Genome completeness was supported by BUSCO analyses, which yielded completeness scores of 97.4% for *E. kansuensis* and 97.5% for *E. juncea* var*. juncea*. The Long Terminal Repeat (LTR) Assembly Index (LAI) scores were 18.75 and 20.53, further indicating high assembly integrity (Table S2). Repetitive elements accounted for 86.37% and 85.32% of the genomes, respectively, dominated by long terminal repeat (LTR) retrotransposons (Table S3). Gene annotation identified 36,754 and 42,167 high-confidence protein-coding genes in *E. kansuensis* and *E. juncea* var*. juncea*, respectively (Tables S4–S6).

**Fig. 1:**
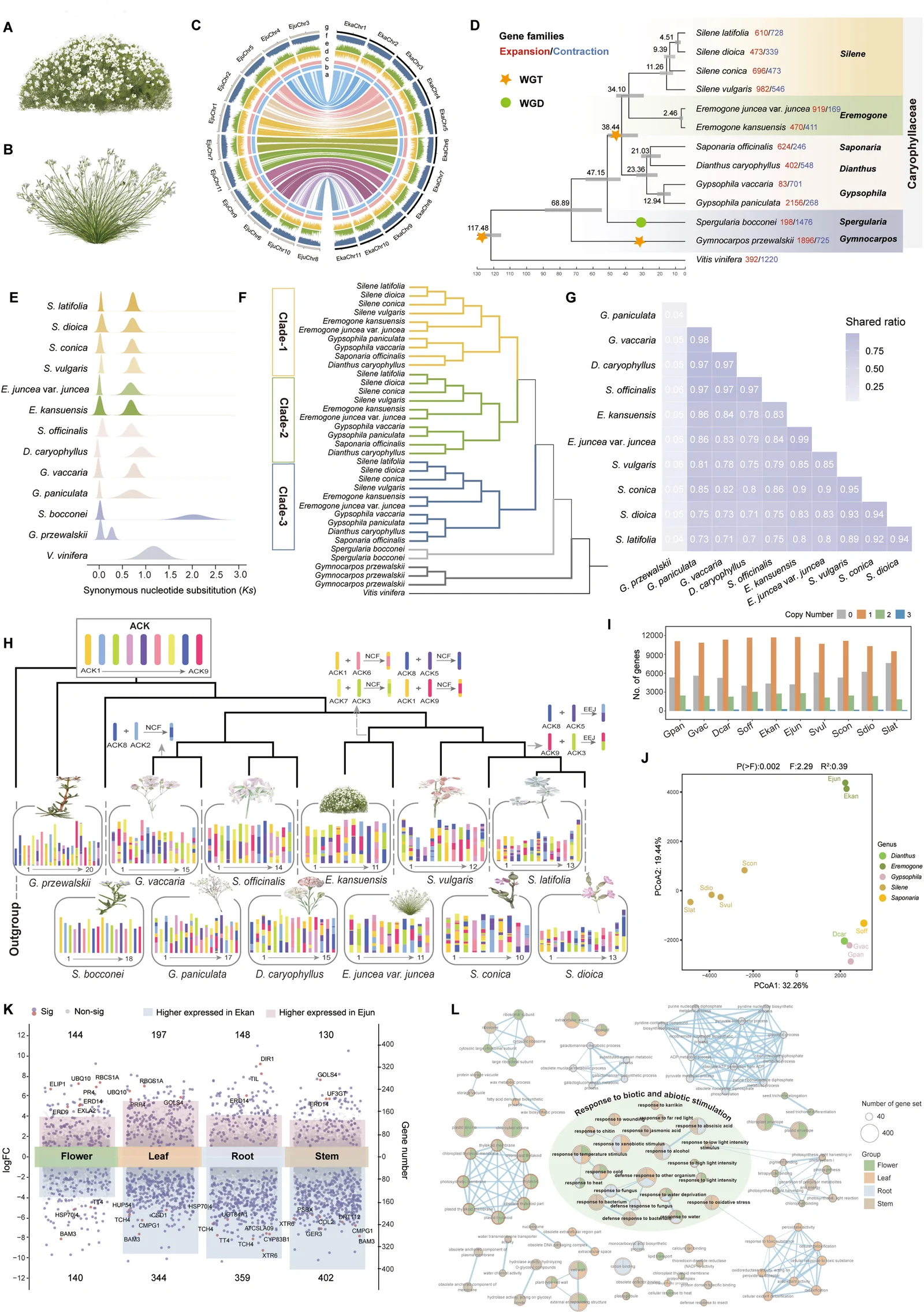
Genome architecture, polyploidy history and transcriptomic divergence in Caryophyllaceae. (A) Morphology of *Eremogone kansuensis*. (B) Morphology of *Eremogone juncea* var. *juncea*. (C) Circos plot showing genome-wide features of *E. kansuensis* and *E. juncea* var. *juncea*. From the inner to the outer circles: (a) identified syntenic blocks between the two genomes; (b) gene density; (c) GC content; (d) density of LTR-Copia elements; (e) density of LTR-Gypsy elements; (f) total LTR density; (g) chromosomes. Values were calculated using 500 kb sliding windows. (D) Phylogenetic relationships among Caryophyllaceae species. Numbers beside each species indicate expanded (red) and contracted (blue) gene families. Whole-genome duplication (WGD) and whole-genome triplication (WGT) events are indicated by green circles and orange stars, respectively. Estimated divergence times (Mya) are shown at internal nodes. A time axis (Mya) is displayed at the bottom of the tree. (E) Distributions of synonymous substitution rates (*Ks*) for collinear gene pairs in each species. (F) Phylogeny reconstructed from collinear gene trees. (G) Heatmap showing the proportion of shared phylogenetic branches among species based on collinear gene trees. (H) Projection of the reconstructed ancestral karyotype (ACK1–ACK9) onto 12 extant Caryophyllaceae species. Colored bars indicate contributions from different ancestral chromosomes. Shared chromosomal fusion events are annotated above the corresponding lineages. (I) Gene copy-number retention patterns of post-γ genes across ten Caryophyllaceae species. Copy numbers were inferred based on ancestral karyotype (ACK) blocks, and bars show the number of genes retained as zero, one, two, or three copies per ancestral locus. (J) Principal component analysis (PCoA) of species-level copy-number profiles based on Manhattan distances. Shapes denote genus identity, and colors indicate major clades. *P*-values were calculated using permutational multivariate analysis of variance (PERMANOVA). (K) Scatter plots of log_2_ fold change (logFC) values of DEGs in each tissue, with gene which has high expression in *E. juncea* var. *juncea* (above side, purple background) and in *E.kansuensis* (below side, blue background). DEGs with significant expression differences are shown in red and purple; selected genes with top10 |logFC| are labeled with homolog of *Arabidopsis*. Numbers above and below each panel indicate the number of genes showed significantly higher expression in *E. kansuensis* and *E. juncea* var. *juncea,* respectively. (L) GO-term enrichment network of DEGs across four tissues. Nodes represent enriched GO terms, colored by tissue type and sized by gene set size. Edges represent functional similarity between GO terms.

To clarify evolutionary relationships within Caryophyllaceae, we reconstructed a nuclear phylogeny using 272 single-copy genes from twelve representative species, with *Vitis vinifera* as the outgroup (Fig. 1D). The resulting nuclear phylogeny recovered a well-supported topology within Caryophyllaceae, resolving *Gymnocarpos* and *Spergularia* successively sister to the remaining five genera, in which *Silene* and *Eremogone* formed a sister relationship, whereas *Saponaria* and *Dianthus* grouped together and were placed alongside *Gypsophila*. The nuclear topology was also consistent with previously published plastome-based phylogenies of Caryophyllaceae (Yang et al., 2024).

Previous studies have suggested that Caryophyllaceae has experienced multiple ancient whole-genome duplication events, yet the ploidy levels and timing of these events remain incompletely resolved, and the number of genome duplication rounds across lineages is still debated (Feng et al., 2024). To characterize the polyploidization history of Caryophyllaceae, we examined intra-species synonymous substitution rate (*Ks*) distributions across all sampled genomes. In contrast to grape (*Vitis vinifera*), which exhibits a single *Ks* peak corresponding to the ancestral γ event, all Caryophyllaceae species showed bimodal *Ks* distributions, indicating two distinct genome-wide duplication signals. In *Silene, Eremogone, Saponaria, Dianthu*s, and *Gypsophila*, the first *Ks* peak was consistently located at low values, whereas the second peak was concentrated within a narrow range between 0.72 and 1.00. In early-diverging lineages, *Ks* peak positions were more variable, with *Spergularia bocconei* showing peaks at 0.065 and 2.08 and *Gymnocarpos przewalskii* showing peaks at 0.073 and 0.31 (Fig. 1E and Table S7).

To resolve the structural nature of these duplication signals, we further performed intra-genomic dot-plot analyses using grape (*Vitis vinifera*) as a reference. Species from *Silene, Eremogone, Dianthus, Saponaria, Gypsophila*, and *Gymnocarpos* exhibited a 1:3 syntenic relationship with grape (Figs. S3–S5), indicating that these lineages experienced the ancestral γ triplication together with an additional post-γ whole-genome triplication (WGT). In contrast, *Spergularia bocconei* displayed a 1:2 syntenic relationship with grape (Fig. S5), consistent with a post-γ WGD. Phylogenetic analyses of collinear gene sets confirmed that the post-γ whole-genome triplication (WGT) is shared by five core Caryophyllaceae genera (*Silene, Eremogone*, *Dianthus*, *Saponaria*, and *Gypsophila*), whereas *Gymnocarpos* exhibited limited branch sharing and a distinct phylogenetic placement (Fig. 1F and G), consistent with an independent lineage-specific triplication. We therefore designate the shared post-γ WGT in the five core genera as the α event.

We reconstructed the ancestral karyotype of Caryophyllaceae (ACK) by applying the WGDI pipeline (Sun et al., 2022) to pairwise synteny analyses of all publicly available Caryophyllaceae genomes included in this study. Conserved chromosome-like synteny blocks (CLSBs) were identified across species, and those consistently preserved as complete, gapless units in distantly related lineages were recognized as ancestral protochromosomes. For each protochromosome, the extant chromosome exhibiting the strongest cross-species collinearity was selected as the ACK candidate. Collinear genes between each candidate and the corresponding chromosomes from other species were then integrated to define the final ACK. This reconstruction yielded an ACK composed of nine ancestral chromosomes (ACK1–ACK9). When compared with extant Caryophyllaceae genomes, each ACK chromosome showed continuous syntenic correspondence without detectable collinearity gaps, supporting that the reconstructed ACK represents, under current sampling, the conserved chromosomal framework of the family (Figs. S6–S10).

Based on the reconstructed ACK, shared chromosomal fusion events were identified within individual Caryophyllaceae lineages (Figs. 1H and S11). In *Silene*, two shared fusion events were detected, involving fusions between ACK8 and ACK5 and between ACK9 and ACK3, both formed through end-to-end joining (EEJ). In *Gypsophila*, a shared fusion between ACK8 and ACK2 was consistently observed in both *Gypsophila vaccaria* and *Gypsophila paniculata*, corresponding to a nested chromosome fusion (NCF). In *Eremogone*, four shared fusion events were detected, all corresponding to NCF, including fusions between ACK1 and ACK6, ACK8 and ACK5, ACK7 and ACK3, and ACK1 and ACK9. Together, these lineage-specific chromosomal fusion patterns support the monophyly of the corresponding genera.

To investigate the evolutionary fate of duplicate genes following the shared α event in five Caryophyllaceae genera, we analyzed gene retention patterns based on the ACK. Most of ACK genes have been retained as single copies across the selected ten species. This widespread gene loss indicates that the genomes have undergone extensive diploidization following the α event (Fig. 1I). Moreover, pairwise comparisons of the proportions of genes retained in different copy-number categories (one-, two-, and three-copy) among the studied species showed no significant differences (*P* > 0.05, chisq. test), suggesting a conserved global pattern of gene fractionation across the lineage. However, when we examined whether different lineages tended to retain the same sets of genes in identical copy numbers, we found a significant association between gene retention patterns and divergence time (PERMANOVA, *P* = 0.002, R^2^ = 0.39) (Fig. 1J). For instance, species from the same genus clustered together, and the three genera (*Saponaria, Dianthus, Gypsophlla*) species with a divergence time around 21.03–23.36 million years ago (Mya) were also clustered together.

Given the conserved copy-number retention patterns observed across the ten species, we further examined functional retention biases in the closely related species pair *Eremogone kansuensis* and *E. juncea* var*. juncea*. GO enrichment analyses were performed for retained genes in different copy-number categories in both species (Tables S8–S13). Genes retained as single or two copies exhibited broadly similar enrichment patterns between the two species and were mainly associated with fundamental cellular and developmental functions. In contrast, genes retained in three copies showed clearer species-specific functional tendencies, suggesting potential divergence in their contribution to local environmental adaptation (Figs. S12 and S13). In *E. kansuensis*, three-copy genes were notably enriched in abiotic stress-related processes, including *response to cold* and *response to temperature stimulus*, together with cytoskeleton-associated functions. By comparison, three-copy genes in *E. juncea* var*. juncea* were primarily associated with general cellular and metabolic processes and showed relatively limited enrichment of stress-related terms (Tables S12–S13).

Differential gene expression is associated with phenotypic diversification and environmental adaptation both within and across species. To uncover mechanisms correlated with high-altitude adaptation in *Eremogone kansuensis*, we performed comparative transcriptomic analyses based on 19,448 syntenic gene pairs identified between *E. kansuensis* and *E. juncea* var*. juncea* across four tissues (flower, leaf, root, and stem). PCA revealed a clear species-level separation, with samples clustering primarily by species rather than by tissue type (Fig. S14). Samples from different tissues within the same species were more similar to each other than those from the same tissue across species along PC1. All tissues showed consistent clustering patterns between species, except for stem samples, which displayed partial separation along PC2. This species-dominated clustering indicates pronounced interspecific divergence in transcriptional regulation between the two closely related species, consistent with recent large-scale evolutionary transcriptomic analyses (Schuster et al., 2026).

We next identified differentially expressed syntenic gene pairs (DEGs) between *Eremogone kansuensis* and *E. juncea* var*. juncea* (See Methods). A total of 284, 541, 507, and 532 gene pairs were detected as differentially expressed in flower, leaf, root, and stem tissues, respectively (FDR < 0.05) (Fig. 1K). Across all tissues, 1130 syntenic gene pairs were identified as differentially expressed in at least one tissue (Fig. S15). Notably, 463 of these DEGs were retained as single-copy after the α event, whereas 287 were retained as multiple copies. Compared with the background distribution of all syntenic gene pairs (8807 single-copy and 4507 multi-copy genes), the copy-number composition of DEGs was significantly different (*P* < 0.05, chisq. test), indicating that genes retained in multiple copies tended to show expression divergence more frequently. This pattern suggests that the α event provided additional genetic resources for subsequent adaptive divergence, while natural selection may have further promoted expression divergence between *E. kansuensis* and *E. juncea* var*. juncea*. Moreover, to explore whether the four shared nested chromosome fusion (NCF) events after the α event in *E. kansuensis* and *E. juncea* var*. juncea* were associated with biased gene retention or stress-related divergence, we analyzed flanking genes from fusion points in both species. No significant copy-retention difference between genomic background and fusion regions was detected in either species (Table S14; chisq. test, *P* > 0.05). Furthermore, 31 gene pairs in the flanking regions overlapped with the 1130 DEGs. Several *Arabidopsis thaliana* homologs of these pairs are known to be associated with cold, dehydration, and salt stress responses, including *CCR2*, *TCH4*, *RD19*, and *SWEET12*. This suggests that these regions may harbor candidate genes with potential relevance to environmental adaptation.

To further explore the potential functions of these DEGs, GO enrichment analysis was performed, which revealed enrichment of several functions associated with multiple environmental responses, including temperature and water stress (e.g., *response to cold*, *temperature stimulus*, and *water deprivation*), light-related responses (e.g., *response to high light intensity*), redox processes (e.g., *response to oxidative stress*), and hormone-related responses (e.g., *response to abscisic acid*). In addition, several enriched GO terms were associated with photosynthesis and chloroplast functions as well as cell wall and extracellular structure components. Consistent patterns were also observed in KEGG pathways, including pathways such as flavonoid biosynthesis, which is commonly associated with photoprotection and oxidative stress responses (Figs. S16 and S17; Tables S15–S19). The GO term network further revealed the functional consistency among the DEG across tissues. A large proportion of enriched terms formed a highly connected cluster associated with responses to biotic and abiotic stimuli, which were shared across all four tissues (Fig. 1L). In addition, several tissue-specific modules were observed, such as chloroplast- and photosynthesis-related GO clusters predominantly enriched in leaves, as well as cuticle formation and cell wall modification terms more prominent in stems (Fig. 1L). Together, these patterns indicate coordinated functional divergence across multiple stress-responsive and metabolic pathways between the two species.

We next examined which differentially expressed syntenic gene pairs were associated with known stress–response processes by integrating curated functional resources. Using *Arabidopsis thaliana* homologs as functional references (see Methods), we queried the STIFDB2 database and identified 173 DEGs directly linked to cold, oxidative, or UV-B responses. Representative candidates included *ELIP1*, *ERD14, ERD9*, *GOLS4*, and *TCH4* (cold-related), *CSD1* (oxidative stress-related), and *UF3GT* (UV–B-related) (Figs. 1K and S18). Notably, among the 29 differentially expressed gene pairs retained in three copies, one gene encoding S-adenosylmethionine synthase (SAMS) showed consistently higher expression across all three copies in the root tissue of *E. kansuensis*. Given that SAMS are involved with responses to multiple abiotic stresses, this pattern suggests a potential role for SAMS in regulatory divergence related to high-altitude adaptation in *E. kansuensis*.

Putative regulatory networks for cold, oxidative, and UV-B responses were reconstructed based on experimentally supported interactions reported in *Arabidopsis* to examine the functional associations of these stress-related genes (Fig. S19). The cold-response network included multiple gene families involved in known stress-signaling pathways, such as *ERD* (Wu et al., 2023), *ELIP* (Hayami et al., 2015), and *GOLS* (Taji et al., 2002), whereas the oxidative and UV-B networks were comparatively more concise because fewer homologous DEGs were detected. These networks provide a framework for interpreting how the identified DEGs may participate in stress–response pathways potentially relevant to high-altitude environments.

In summary, we generated high-quality chromosome-scale genome assemblies for the alpine species *Eremogone kansuensis* and its low-altitude relative *E. juncea* var*. juncea*, and estimated a recent divergence (∼2.46 Mya) between them. Using genome-wide synteny, we reconstructed the ancestral Caryophyllaceae karyotype (ACK, *n* = 9), providing a reference framework for comparative genomic analyses. We further identified a shared whole-genome triplication (WGT) event (α) across *Eremogone* and four additional Caryophyllaceae genera, accompanied by conserved patterns of gene retention. Comparative transcriptome analyses across four tissues revealed extensive regulatory divergence between the two species, with differentially expressed genes enriched in pathways associated with multiple environmental responses. Several representative stress-related genes, including *ELIP1*, *ERD14*, *GOLS4*, and *UF3GT*, were identified among these candidates. Together, these findings integrate genome evolution and regulatory divergence, providing a genomic framework for understanding molecular processes potentially associated with high-altitude adaptation in *E. kansuensis*. More broadly, these findings provide insights into how genome duplication and subsequent regulatory divergence may be linked to ecological differentiation in plants.

## CRediT authorship contribution statement

**Lizuiyue Li**: Writing - Original Draft, Software, Investigation, Visualization, Formal analysis, Data Curation. **Nannan Zhang**: Visualization. **Liqiang Hou**: Formal analysis, Data Curation. **Xiaozhu Guo**: Formal analysis, Data Curation. **Huizhe Feng**: Resources. **Yongzhi Yang**: Writing - Review & Editing, Conceptualization. **Bin Tian**: Writing - Review & Editing, Conceptualization.

## Data availability

Whole genome sequencing reads and RNA-seq reads are available in the National Genomics Data Center (NGDC) database under BioProject accession numbers PRJCA056851 and PRJCA056867. The genome assemblies and gene annotation files for *Eremogone kansuensis* and *Eremogone juncea* var. *juncea* have been deposited in Figshare under https://doi.org/10.6084/m9.figshare.32103184.

## Declaration of competing interest

The authors declare that they have no known competing financial interests or personal relationships that could have appeared to influence the work reported in this paper.
