# Supplementary material for: Two Eremogone (Caryophyllaceae) genomes provide insights into alpine adaptation
Source: Plant Divers. 2026 May 21;48(5):1064–8. doi: 10.1016/j.pld.2026.05.008 (PMC13615786; doi:10.1016/j.pld.2026.05.008)
Supplement: Supplementary file 1 [file mmc1.docx]

**Two *Eremogone* (Caryophyllaceae) genomes provide insights into alpine adaptation**

Lizuiyue Li (李李醉乐)^a,b^, Nannan Zhang (张楠楠)^c^, Liqiang Hou (侯利强)^c^, Xiaozhu Guo (郭晓竹)^c^, Huizhe Feng (冯慧喆)^d^, Yongzhi Yang (杨勇志)^c^*, Bin Tian (田斌)^a,b,^*

^a^Yunnan Key Laboratory of Plateau Wetland Conservation, Restoration and Ecological Services, Southwest Forestry University, Kunming 650224, China

^b^Shangri-La Potatso National Park Bita Lake Plateau Wetland Ecosystem Observation and Research Station of Yunnan Province, Kunming 650233, China

^c^State Key Laboratory of Herbage Improvement and Grassland Agro-Ecosystems, College of Ecology, Lanzhou University, Lanzhou 730000, China

^d^College of Life Sciences, Zaozhuang University, Zaozhuang 277160, China

*Corresponding authors:

Yongzhi Yang, yangyz@lzu.edu.cn;

Bin Tian, tianbin@swfu.edu.cn

**SUPPORTING DATA**

**Figure. S1** Hi-C interaction heatmaps of *Eremogone kansuensis* and *Eremogone juncea* var*. juncea*.

**Figure. S2** 31-mer distribution profiles of the genomes of *Eremogone kansuensis* and *Eremogone juncea* var*. juncea.*

**Figure. S3** Synteny dot plots between Caryophyllaceae species and *Vitis vinifera*.

Synteny dot plots comparing *Gypsophila paniculata*, *G. vaccaria*, *Saponaria officinalis*, and *Dianthus caryophyllus* with *Vitis vinifera*. Red dots indicate the best homologous gene pairs, while blue dots represent secondary homologs. Light green boxes highlight representative syntenic blocks showing a predominant 1:3 correspondence between *V. vinifera* and Caryophyllaceae chromosomes, consistent with a shared whole-genome triplication in Caryophyllaceae.

**Figure. S4** Synteny dot plots between four *Silene* species and *Vitis vinifera*.

Synteny dot plots comparing *Silene latifolia*, *S. conica*, *S. vulgaris*, and *S. dioica* with *Vitis vinifera*. Red dots represent the most homologous gene pairs, while blue dots indicate secondary homologs. Light green boxes highlight representative syntenic blocks. All four *Silene* species exhibit a consistent 1:3 syntenic relationship with *V. vinifera*, supporting a shared whole-genome triplication in this lineage.

**Figure. S5** Synteny dot plots between Caryophyllaceae species and *Vitis vinifera*.

Synteny dot plots comparing *Eremogone juncea* var. *juncea*, *E. kansuensis*, *Gymnocarpos przewalskii*, and *Spergularia bocconei* with *Vitis vinifera*. Red dots indicate the best homologous gene pairs, and blue dots represent secondary homologs. Light green boxes highlight representative syntenic blocks. *Eremogone* species and *Gymnocarpos* show a predominant 1:3 syntenic correspondence with *V. vinifera*, whereas *S. bocconei* exhibits a 1:2 relationship, consistent with a lineage-specific whole-genome duplication rather than triplication.

**Figure. S6** Synteny dot plots between the ancestral Caryophyllaceae karyotype (ACK) and four Caryophyllaceae species.

Synteny dot plots showing collinear relationships between the ancestral Caryophyllaceae karyotype (ACK) and four Caryophyllaceae species: *Gypsophila paniculata*, *G. vaccaria*, *Saponaria officinalis*, and *Dianthus caryophyllus*. Colored blocks indicate ancestral chromosomes (ACK1–ACK9), and dots represent collinear gene pairs.

**Figure. S7** Synteny dot plots between the ancestral Caryophyllaceae karyotype (ACK) and four Caryophyllaceae species.

Synteny dot plots showing collinear relationships between the ancestral Caryophyllaceae karyotype (ACK) and four Caryophyllaceae species: *Eremogone juncea* var. *juncea, E. kansuensis, Gymnocarpos przewalskii,* and *Spergularia bocconei.* Colored blocks indicate ancestral chromosomes (ACK1–ACK9), and dots represent collinear gene pairs.

**Figure. S8** Synteny dot plots between the ancestral Caryophyllaceae karyotype (ACK) and four Caryophyllaceae species.

Synteny dot plots showing collinear relationships between the ancestral Caryophyllaceae karyotype (ACK) and four Caryophyllaceae species: *Silene latifolia, S. conica, S. vulgaris, and S. dioica.* Colored blocks indicate ancestral chromosomes (ACK1–ACK9), and dots represent collinear gene pairs.

**Figure. S9** Dot-plot analyses of chromosomes showing complete mapping to the ancestral Caryophyllaceae karyotype (ACK).

Dot-plot analyses of chromosomes in representative Caryophyllaceae species that exhibit complete syntenic correspondence to individual ancestral Caryophyllaceae karyotype (ACK) chromosomes. Each panel shows intra-genomic collinearity of chromosomes selected based on full-length mapping to a single ACK chromosome. The continuous diagonal patterns indicate structural integrity and support the completeness of ACK reconstruction.

**Figure. S10** Identification of conserved chromosome-like synteny blocks used for reconstruction of the ancestral Caryophyllaceae karyotype (ACK)

Complete and conserved chromosome-like synteny blocks identified across Caryophyllaceae genomes and used for reconstruction of the ancestral Caryophyllaceae karyotype (ACK). Each block represents an ancestral chromosomal segment retained in collinear form across extant species.

**Figure. S11** Dot-plot evidence supporting the shared chromosomal fusion events identified in Caryophyllaceae.

(A) Four shared chromosomal fusion events identified in *Eremogone*. (B) Two shared chromosomal fusion events identified in *Silene*. (C) One shared chromosomal fusion event identified in *Gypsophila*. Representative chromosome pairs are shown for each inferred fusion event.

**Figure. S12** Functional enrichment of three-copy genes retained after the α event in *Eremogone kansuensis*.

Gene Ontology (GO) enrichment analysis of α event genes retained as three copies in *E. kansuensis*. Enriched GO terms are grouped into biological process (BP), cellular component (CC), and molecular function (MF) categories.

**Figure. S13** Functional enrichment of three-copy genes retained after the α event in *Eremogone juncea* var. *juncea*.

Gene Ontology (GO) enrichment analysis of α event genes retained as three copies in *E. juncea* var. *juncea*. Enriched GO terms are grouped into biological process (BP), cellular component (CC), and molecular function (MF) categories.

**Figure. S14** Principal component analysis (PCA) of transcriptomic profiles in *Eremogone kansuensis* and *E. juncea* var. *juncea*.
PCA was performed based on normalized expression levels of 19,448 syntenic gene pairs across four tissues: flower (F), leaf (L), root (R), and stem (S), with three biological replicates per tissue. Each point represents one biological replicate, colored by species (*E. kansuensis* and *E. juncea* var. *juncea*) and shaped by tissue type. The percentages of variance explained by the first two principal components (PC1 and PC2) are indicated on the axes.

**Figure. S15** Tissue-specific and shared differentially expressed syntenic gene pairs between *Eremogone kansuensis* and *E. juncea* var. *juncea.*

Venn diagram showing the number of differentially expressed synteny gene pairs (DEGs) between *E. kansuensis* and *E. juncea* var. *juncea* across four tissues (flower, leaf, root, and stem).

**Figure. S16** Tissue-specific Gene Ontology enrichment of differentially expressed syntenic gene pairs.

Gene Ontology (GO) enrichment analysis of differentially expressed syntenic gene pairs between *Eremogone kansuensis* and *E. juncea* var. *juncea* across four tissues: stem (S), root (R), flower (F), and leaf (L). Enriched GO terms are shown for the three GO categories, biological process (BP), cellular component (CC), and molecular function (MF). Bars represent the number of genes associated with each GO term, and color intensity indicates enrichment significance (−log₁₀ *P* value). Only significantly enriched terms (FDR < 0.05) are displayed.

**Figure. S17** KEGG pathway enrichment of differentially expressed syntenic gene pairs.

KEGG pathway enrichment analysis was performed for syntenic gene pairs that were differentially expressed between *Eremogone kansuensis* and *E. juncea* var. *juncea* in at least one tissue. The bar length indicates the number of genes enriched in each pathway, and color intensity represents the significance level (–log₁₀ *P* value).

**Figure. S18** Stress-associated differentially expressed gene pairs across tissues.

Circular plot showing differentially expressed syntenic gene pairs associated with cold, oxidative, and UV-B stress responses, based on functional annotation using *Arabidopsis thaliana* homologs and the STIFDB2 database (see Methods). Outer sectors represent tissues (flower, leaf, root, and stem), with numbers indicating the total stress-associated DEGs detected per tissue. Ribbons indicate DEGs shared among tissues. Bars denote the number of DEGs assigned to each stress category. Colors represent stress types: cold (blue), oxidative (red), and UV-B (purple).

**Figure. S19** Transcriptional regulatory networks associated with cold, UV-B, and oxidative stress responses.

Interaction networks of differentially expressed genes (DEGs) under three abiotic stress conditions: cold stress (left), UV-B stress (top right), and oxidative stress (bottom right). Each node represents a gene homologous to *Arabidopsis thaliana*, and edges represent regulatory interactions. Nodes are colored according to differential expression status between *Eremogone kansuensis* and *E. juncea* var. *juncea* . Red nodes indicate significantly differentially expressed genes, whereas blue nodes indicate non-significant genes. Solid edges indicate direct interactions, and dashed edges represent transcription factor (TF)–target regulatory relationships.

**Table S1** Genome assembly statistics of *Eremogone kansuensis* and *E. juncea* var. *juncea*

| **Items** | ***E. kansuensis*** | ***E. juncea* var. *juncea*** |
| --- | --- | --- |
| Total length of contigs (Gb) | 2.43 | 2.09 |
| Number of contigs | 1176 | 1777 |
| Contig N50 (Mb) | 55.18 | 9.81 |
| Total length of chromosome-level assembly (Gb) | 1.82 | 1.83 |
| Scaffold N50 (Mb) | 165.42 | 165.28 |
| Number of chromosomes | 11 | 11 |
| Gap num | 76 | 351 |
| Percentage of anchoring (%) | 90.57 | 87.55 |
| Mean GC content (%) | 39.28 | 38.66 |

**Table S2** Genome quality assessment statistics of *Eremogone kansuensis* and *E. juncea* var. *juncea*

| **Items** | ***E. kansuensis*** | ***E. juncea* var. *juncea*** |
| --- | --- | --- |
| Complete BUSCOs (%) | 97.4 | 97.5 |
| Complete and single-copy BUSCOs (%) | 90.1 | 87.6 |
| Complete and duplicated BUSCOs (%) | 7.2 | 9.9 |
| Fragmented BUSCOs (%) | 0.7 | 1 |
| Missing BUSCOs (%) | 1.9 | 1.5 |
| Fraction of Mapped Data (%) | 99.89 | 99.38 |
| Regions of Coverage >=4x (%) | 98.90 | 98.37 |
| LAI | 18.75 | 20.53 |

**Table S3** Summary of repetitive elements annotation for *Eremogone kansuensis* and *E. juncea* var. *juncea*

|  | ***E. kansuensis*** | | ***E. juncea* var. *juncea*** | |
| --- | --- | --- | --- | --- |
| Repetitive element type | Length (bp) | Percentage (%) in genome | Length (bp) | Percentage (%) in genome |
| SINE | 2331 | 0.021 | 1369 | 0.011 |
| LINE/L1 | 22479273 | 1.235 | 25494471 | 1.394 |
| LINE/Other LINEs | 2976959 | 0.164 | 4787231 | 0.262 |
| LTR/Copia | 107493118 | 5.904 | 129764525 | 7.096 |
| LTR/Gypsy | 475404326 | 26.111 | 426219506 | 23.307 |
| LTR/Other LTR retrotransposons | 712760797 | 39.148 | 746253267 | 40.806 |
| DNA/CMC-EnSpm | 29678554 | 1.63 | 28952000 | 1.583 |
| DNA/hAT-Ac | 7997918 | 0.439 | 3409452 | 0.186 |
| DNA/hAT-Tip100 | 598473 | 0.033 | 684666 | 0.037 |
| DNA/MuDR | 34448163 | 1.892 | 36695737 | 2.007 |
| DNA/PIF-Harbinger | 3329816 | 0.183 | 2781490 | 0.152 |
| DNA/Other DNA transposons | 1066585 | 0.058 | 11054969 | 0.604 |
| Unclassified TEs | 16643279 | 0.914 | 358430 | 0.02 |
| Transposable elements (TEs) total | 1414879592 | 77.711 | 1416457113 | 77.455 |
| Satellite repeats | 11856 | 0.001 | 17151 | 0.001 |
| Simple repeats | 8785634 | 0.483 | 10892096 | 0.596 |
| Small RNA | 3728646 | 0.205 | 4563988 | 0.25 |
| Unclassified repeats | 145102476 | 7.97 | 128381344 | 7.02 |
| Total repeats | 1572508204 | 86.37 | 1560311692 | 85.322 |

**Table S4** Gene structure prediction statistics of *Eremogone kansuensis* and *E. juncea* var. *juncea*

|  | ***E. kansuensis*** | | ***E. juncea* var. *juncea*** | |
| --- | --- | --- | --- | --- |
|  | Only Chromosome | Total | Only Chromosome | Total |
| No. gene Predicted | 36754 | 41187 | 42167 | 49805 |
| Average Gene Length (bp) | 4868.56 | 4702.64 | 4668.96 | 4422.02 |
| Average CDS Length (bp) | 1207.49 | 1224.48 | 1154.87 | 1148.14 |
| Average Exons per Gene | 4.71 | 4.51 | 4.55 | 4.31 |
| Average Exon Length (bp) | 256.26 | 271.08 | 253.6 | 266.21 |
| Average Intron Length (bp) | 986.28 | 988.98 | 1176.73 | 1179.95 |

**Table S5** Summary of functional annotation of gene models in *Eremogone kansuensis* and *E. juncea* var. *juncea*

|  | ***E. kansuensis*** | | ***E. juncea* var. *juncea*** | |
| --- | --- | --- | --- | --- |
|  | Number | Percent (%) | Number | Percent (%) |
| InterPro | 34261 | 93.22 | 38708 | 91.80 |
| GO | 13493 | 36.71 | 15014 | 35.61 |
| iTAK | 3328 | 9.05 | 3539 | 8.39 |
| Total | 34292 | 93.30 | 38789 | 91.99 |

**Table S6** Quality assessment of the gene annotations of *Eremogone kansuensis* and *E. juncea* var. *juncea* using BUSCO

|  | ***E. kansuensis*** | ***E. juncea* var. *juncea*** |
| --- | --- | --- |
| Complete BUSCOs (%) | 97.4 | 97.7 |
| Complete and single-copy BUSCOs (%) | 90.0 | 88.4 |
| Complete and duplicated BUSCOs (%) | 7.4 | 9.3 |
| Fragmented BUSCOs (%) | 0.6 | 0.3 |
| Missing BUSCOs (%) | 2.0 | 2.0 |
| Database used | embryophyta_odb10 | embryophyta_odb10 |

**Table S7** Estimated *Ks* peak values inferred from fitted distributions of collinear gene pairs in the analyzed species

| **Species** | **Peak 1 (*Ks*)** | **Peak 2 (*Ks*)** |
| --- | --- | --- |
| *Eremogone kansuensis* | 0.0594 | 0.7346 |
| *Eremogone juncea* var. *juncea* | 0.0507 | 0.7259 |
| *Silene latifolia* | 0.0848 | 0.7754 |
| *Silene vulgaris* | 0.0916 | 0.7946 |
| *Silene dioica* | 0.1004 | 0.7673 |
| *Silene conica* | 0.0666 | 0.7605 |
| *Saponaria officinalis* | 0.1142 | 0.7375 |
| *Dianthus caryophyllus* | 0.0101 | 0.9559 |
| *Gypsophila paniculata* | 0.0516 | 0.7645 |
| *Gypsophila vaccaria* | 0.0294 | 0.8037 |
| *Spergularia bocconei* | 0.0656 | 1.5009 |
| *Gymnocarpos przewalskii* | 0.0730 | 0.3184 |
| *Vitis vinifera* |  | 1.2211 |

**Table S8** Gene Ontology (GO) enrichment analysis of one-copy genes retained in *Eremogone juncea* var. *juncea*.

| **ID** | **Term Name** | **p. adjust** | **Category** |
| --- | --- | --- | --- |
| GO:0000151 | Ubiquitin ligase complex | 2.37E-06 | CC |
| GO:0031461 | Cullin-RING ubiquitin ligase complex | 9.26E-05 | CC |
| GO:0006281 | DNA repair | 3.37E-04 | BP |
| GO:0031977 | Thylakoid lumen | 0.002163 | CC |
| GO:0006974 | DNA damage response | 0.002364 | BP |
| GO:0009767 | Photosynthetic electron transport chain | 0.002364 | BP |
| GO:0009657 | Plastid organization | 0.002603 | BP |
| GO:0004497 | Monooxygenase activity | 0.006408 | MF |

**Table S9** Gene Ontology (GO) enrichment analysis of one-copy genes retained in *Eremogone kansuensis.*

| **ID** | **Term Name** | **p. adjust** | **Category** |
| --- | --- | --- | --- |
| GO:0006974 | DNA damage response | 9.08E-07 | BP |
| GO:0006281 | DNA repair | 6.03E-06 | BP |
| GO:0007059 | Chromosome segregation | 1.92E-05 | BP |
| GO:0009657 | Plastid organization | 4.41E-05 | BP |
| GO:0007049 | Cell cycle | 4.41E-05 | BP |
| GO:0098813 | Nuclear chromosome segregation | 4.41E-05 | BP |
| GO:0000819 | Sister chromatid segregation | 4.78E-05 | BP |
| GO:0022402 | Cell cycle process | 4.94E-05 | BP |
| GO:0000151 | Ubiquitin ligase complex | 7.52E-04 | CC |
| GO:0010598 | NAD(P)H dehydrogenase complex (plastoquinone) | 8.00E-04 | CC |
| GO:0044798 | Transcription regulator complex | 8.00E-04 | CC |
| GO:0006261 | DNA-templated DNA replication | 9.92E-04 | BP |
| GO:0000280 | Nuclear division | 9.92E-04 | BP |
| GO:0005694 | Chromosome | 0.001636 | CC |
| GO:0090575 | RNA polymerase II transcription regulator complex | 0.001766 | CC |
| GO:0000278 | Mitotic cell cycle | 0.0018 | BP |
| GO:0048285 | Organelle fission | 0.00211 | BP |
| GO:0009658 | Chloroplast organization | 0.00211 | BP |
| GO:0004536 | DNA nuclease activity | 0.002421 | MF |
| GO:0006289 | Nucleotide-excision repair | 0.002486 | BP |
| GO:0048509 | Regulation of meristem development | 0.002486 | BP |
| GO:0005667 | Transcription regulator complex | 0.002638 | CC |
| GO:0042646 | Plastid nucleoid | 0.003279 | CC |
| GO:0140014 | Mitotic nuclear division | 0.003526 | BP |
| GO:0000070 | Mitotic sister chromatid segregation | 0.003526 | BP |
| GO:0006352 | DNA-templated transcription initiation | 0.003899 | BP |
| GO:0000079 | Regulation of cyclin-dependent protein serine/threonine kinase activity | 0.004107 | BP |
| GO:1904029 | Obsolete regulation of cyclin-dependent protein kinase activity | 0.004107 | BP |
| GO:1905393 | Plant organ formation | 0.004598 | BP |
| GO:0016853 | Isomerase activity | 0.005032 | MF |
| GO:0008610 | Lipid biosynthetic process | 0.005348 | BP |
| GO:0044272 | Sulfur compound biosynthetic process | 0.007623 | BP |
| GO:0007062 | Sister chromatid cohesion | 0.007623 | BP |
| GO:0006260 | DNA replication | 0.00835 | BP |
| GO:1903047 | Mitotic cell cycle process | 0.008774 | BP |

**Table S10** Gene Ontology (GO) enrichment analysis of two-copy genes retained in *Eremogone juncea* var. *juncea* (top 30 terms ranked by adjusted *P* value).

| **ID** | **Term Name** | **p. adjust** | **Category** |
| --- | --- | --- | --- |
| GO:0016049 | Cell growth | 4.28E-06 | BP |
| GO:0010200 | Response to chitin | 8.57E-06 | BP |
| GO:0048589 | Developmental growth | 1.39E-05 | BP |
| GO:0060560 | Developmental growth involved in morphogenesis | 2.86E-05 | BP |
| GO:0000325 | Plant-type vacuole | 4.54E-05 | CC |
| GO:0009826 | Unidimensional cell growth | 4.73E-05 | BP |
| GO:0009723 | Response to ethylene | 4.73E-05 | BP |
| GO:0044212 | Transcription cis-regulatory region binding | 6.94E-05 | MF |
| GO:0001067 | Transcription regulatory region nucleic acid binding | 6.94E-05 | MF |
| GO:0004842 | Ubiquitin-protein transferase activity | 6.94E-05 | MF |
| GO:0070696 | Transmembrane receptor protein serine/threonine kinase binding | 1.18E-04 | MF |
| GO:0019787 | Ubiquitin-like protein transferase activity | 1.36E-04 | MF |
| GO:0009733 | Response to auxin | 1.36E-04 | BP |
| GO:0045926 | Negative regulation of growth | 4.08E-04 | BP |
| GO:0010052 | Guard cell differentiation | 4.08E-04 | BP |
| GO:0048580 | Regulation of post-embryonic development | 4.08E-04 | BP |
| GO:0043622 | Cortical microtubule organization | 4.17E-04 | BP |
| GO:0031122 | Cytoplasmic microtubule organization | 4.17E-04 | BP |
| GO:0010558 | Negative regulation of macromolecule biosynthetic process | 4.76E-04 | BP |
| GO:2000241 | Regulation of reproductive process | 0.001229 | BP |
| GO:0010091 | Trichome branching | 0.001277 | BP |
| GO:0009860 | Pollen tube growth | 0.001283 | BP |
| GO:2000012 | Regulation of auxin polar transport | 0.001283 | BP |
| GO:0009932 | Cell tip growth | 0.001554 | BP |
| GO:0009705 | Plant-type vacuole membrane | 0.001714 | CC |
| GO:0048588 | Developmental cell growth | 0.002042 | BP |
| GO:0032352 | Positive regulation of hormone metabolic process | 0.002042 | BP |
| GO:0048511 | Rhythmic process | 0.00208 | BP |
| GO:0045177 | Apical part of cell | 0.002085 | CC |
| GO:0030865 | Cortical cytoskeleton organization | 0.002089 | BP |
| GO:0048868 | Pollen tube development | 0.002313 | BP |

**Table S11** Gene Ontology (GO) enrichment analysis of two-copy genes retained in *Eremogone kansuensis* (top 30 terms ranked by adjusted *P* value)*.*

| **ID** | **Term Name** | **p. adjust** | **Category** |
| --- | --- | --- | --- |
| GO:0000902 | Cell morphogenesis | 3.46E-08 | BP |
| GO:0016049 | Cell growth | 4.19E-08 | BP |
| GO:0009826 | Unidimensional cell growth | 4.19E-08 | BP |
| GO:0048589 | Developmental growth | 1.19E-07 | BP |
| GO:0000325 | Plant-type vacuole | 5.31E-07 | CC |
| GO:0060560 | Developmental growth involved in morphogenesis | 1.48E-06 | BP |
| GO:0045177 | Apical part of cell | 1.73E-06 | CC |
| GO:0016324 | Apical plasma membrane | 1.96E-06 | CC |
| GO:0009705 | Plant-type vacuole membrane | 1.96E-06 | CC |
| GO:0019899 | Enzyme binding | 3.95E-06 | MF |
| GO:0001067 | Transcription regulatory region nucleic acid binding | 6.28E-06 | MF |
| GO:0044212 | Transcription cis-regulatory region binding | 6.28E-06 | MF |
| GO:0009932 | Cell tip growth | 8.45E-06 | BP |
| GO:0048468 | Cell development | 8.74E-06 | BP |
| GO:0010052 | Guard cell differentiation | 4.98E-05 | BP |
| GO:0010558 | Negative regulation of macromolecule biosynthetic process | 4.98E-05 | BP |
| GO:0007029 | Endoplasmic reticulum organization | 1.16E-04 | BP |
| GO:0009733 | Response to auxin | 1.70E-04 | BP |
| GO:0009860 | Pollen tube growth | 2.36E-04 | BP |
| GO:0010200 | Response to chitin | 2.36E-04 | BP |
| GO:0045892 | Negative regulation of DNA-templated transcription | 2.36E-04 | BP |
| GO:1902679 | Negative regulation of RNA biosynthetic process | 2.36E-04 | BP |
| GO:2000241 | Regulation of reproductive process | 2.36E-04 | BP |
| GO:0016192 | Vesicle-mediated transport | 2.77E-04 | BP |
| GO:0051253 | Negative regulation of RNA metabolic process | 2.77E-04 | BP |
| GO:0009890 | Negative regulation of biosynthetic process | 2.81E-04 | BP |
| GO:0048511 | Rhythmic process | 3.06E-04 | BP |
| GO:0010256 | Endomembrane system organization | 3.06E-04 | BP |
| GO:0009723 | Response to ethylene | 4.21E-04 | BP |
| GO:0008283 | Cell population proliferation | 7.33E-04 | BP |

**Table S12** Gene Ontology (GO) enrichment analysis of three-copy genes retained in *Eremogone juncea* var. *juncea* (top 30 terms ranked by adjusted *P* value).

| **ID** | **Term Name** | **p. adjust** | **Category** |
| --- | --- | --- | --- |
| GO:0022626 | Cytosolic ribosome | 1.96E-12 | CC |
| GO:0022625 | Cytosolic large ribosomal subunit | 6.81E-12 | CC |
| GO:0044391 | Ribosomal subunit | 1.42E-11 | CC |
| GO:0015934 | Large ribosomal subunit | 3.73E-10 | CC |
| GO:0003735 | Structural constituent of ribosome | 1.94E-09 | MF |
| GO:0015450 | Protein-transporting atpase activity | 6.93E-07 | MF |
| GO:0005742 | Mitochondrial outer membrane translocase complex | 2.63E-06 | CC |
| GO:0008320 | Protein transmembrane transporter activity | 3.29E-06 | MF |
| GO:0022884 | Macromolecule transmembrane transporter activity | 3.29E-06 | MF |
| GO:0098799 | Outer mitochondrial membrane protein complex | 4.26E-06 | CC |
| GO:0000788 | Nucleosome | 3.83E-04 | CC |
| GO:0022627 | Cytosolic small ribosomal subunit | 5.52E-04 | CC |
| GO:2001257 | Regulation of cation channel activity | 0.001003 | BP |
| GO:0009225 | Nucleotide-sugar metabolic process | 0.00106 | BP |
| GO:0046777 | Protein autophosphorylation | 0.00106 | BP |
| GO:0009065 | Glutamine family amino acid catabolic process | 0.00106 | BP |
| GO:0022898 | Regulation of transmembrane transporter activity | 0.00106 | BP |
| GO:0032409 | Regulation of transporter activity | 0.00106 | BP |
| GO:0032412 | Regulation of monoatomic ion transmembrane transporter activity | 0.00106 | BP |
| GO:1904680 | Peptide transmembrane transporter activity | 0.001537 | MF |
| GO:0046686 | Response to cadmium ion | 0.001807 | BP |
| GO:0042776 | Proton motive force-driven mitochondrial ATP synthesis | 0.001807 | BP |
| GO:0001228 | DNA-binding transcription activator activity, RNA polymerase II-specific | 0.001896 | MF |
| GO:0006839 | Mitochondrial transport | 0.00204 | BP |
| GO:0001666 | Response to hypoxia | 0.00204 | BP |
| GO:0003690 | Double-stranded DNA binding | 0.002144 | MF |
| GO:0005985 | Sucrose metabolic process | 0.002207 | BP |
| GO:0045010 | Actin nucleation | 0.002207 | BP |
| GO:1901606 | Alpha-amino acid catabolic process | 0.002718 | BP |
| GO:0042887 | Amide transmembrane transporter activity | 0.003255 | MF |

**Table S13** Gene Ontology (GO) enrichment analysis of three-copy genes retained in *Eremogone kansuensis* (top 30 terms ranked by adjusted *P* value)*.*

| **ID** | **Term Name** | **p. adjust** | **Category** |
| --- | --- | --- | --- |
| GO:0015450 | Protein-transporting atpase activity | 1.73E-07 | MF |
| GO:0008320 | Protein transmembrane transporter activity | 3.64E-07 | MF |
| GO:0022884 | Macromolecule transmembrane transporter activity | 3.64E-07 | MF |
| GO:0008154 | Actin polymerization or depolymerization | 6.27E-07 | BP |
| GO:0000981 | DNA-binding transcription factor activity, RNA polymerase II-specific | 9.10E-07 | MF |
| GO:0007015 | Actin filament organization | 3.54E-06 | BP |
| GO:0030042 | Actin filament depolymerization | 6.08E-06 | BP |
| GO:0097435 | Supramolecular fiber organization | 1.23E-05 | BP |
| GO:0003779 | Actin binding | 3.26E-05 | MF |
| GO:0030036 | Actin cytoskeleton organization | 4.12E-05 | BP |
| GO:0000788 | Nucleosome | 6.16E-05 | CC |
| GO:0051261 | Protein depolymerization | 6.50E-05 | BP |
| GO:0051258 | Protein polymerization | 7.16E-05 | BP |
| GO:0051015 | Actin filament binding | 1.70E-04 | MF |
| GO:0030029 | Actin filament-based process | 1.77E-04 | BP |
| GO:0009631 | Cold acclimation | 2.30E-04 | BP |
| GO:0022625 | Cytosolic large ribosomal subunit | 3.03E-04 | CC |
| GO:0000790 | Chromatin | 3.03E-04 | CC |
| GO:1904680 | Peptide transmembrane transporter activity | 3.09E-04 | MF |
| GO:0022626 | Cytosolic ribosome | 3.15E-04 | CC |
| GO:0016903 | Oxidoreductase activity, acting on the aldehyde or oxo group of donors | 3.36E-04 | MF |
| GO:0009225 | Nucleotide-sugar metabolic process | 4.50E-04 | BP |
| GO:0051262 | Protein tetramerization | 5.03E-04 | BP |
| GO:0000786 | Nucleosome | 7.11E-04 | CC |
| GO:0009409 | Response to cold | 7.30E-04 | BP |
| GO:0001067 | Transcription regulatory region nucleic acid binding | 9.96E-04 | MF |
| GO:0044212 | Transcription cis-regulatory region binding | 9.96E-04 | MF |
| GO:0001228 | DNA-binding transcription activator activity, RNA polymerase II-specific | 9.96E-04 | MF |
| GO:0042887 | Amide transmembrane transporter activity | 0.001643 | MF |
| GO:0032993 | Protein-DNA complex | 0.001897 | CC |

**Table S14** Chromosomal fusion breakpoints in *Eremogone kansuensis* and *E. juncea* var. *juncea*

| **Species** | **Chromosome** | **Fusion Event** | **Breakpoint 1 Position (bp)** | **Breakpoint 2 Position (bp)** | **Fusion Type** |
| --- | --- | --- | --- | --- | --- |
| *E. kansuensis* | Chr01 | Fusion 1 | 730 | 831 | NCF |
| *E. juncea* var. *juncea* | Chr03 | Fusion 1 | 973 | 2975 | NCF |
| *E. kansuensis* | Chr10 | Fusion 2 | 743 | 2238 | NCF |
| *E. juncea* var. *juncea* | Chr10 | Fusion 2 | 847 | 2740 | NCF |
| *E. kansuensis* | Chr03 | Fusion 3 | 915 | 2654 | NCF |
| *E. juncea* var. *juncea* | Chr05 | Fusion 3 | 1018 | 2748 | NCF |
| *E. kansuensis* | Chr07 | Fusion 4 | 790 | 2618 | NCF |
| *E. juncea* var. *juncea* | Chr11 | Fusion 4 | 881 | 3067 | NCF |

**Table S15** Gene Ontology (GO) enrichment analysis of differentially expressed syntenic gene pairs between *Eremogone kansuensis* and *E. juncea* var. *juncea* in leaf (significantly enriched terms, FDR < 0.05).

| **ID** | **Term Name** | **p. adjust** | **Category** |
| --- | --- | --- | --- |
| GO:0009534 | Chloroplast thylakoid | 6.02E-08 | CC |
| GO:0031976 | Plastid thylakoid | 6.02E-08 | CC |
| GO:0009535 | Chloroplast thylakoid membrane | 2.89E-07 | CC |
| GO:0055035 | Plastid thylakoid membrane | 2.89E-07 | CC |
| GO:0042651 | Thylakoid membrane | 4.12E-07 | CC |
| GO:0034357 | Photosynthetic membrane | 4.12E-07 | CC |
| GO:0009579 | Thylakoid | 4.12E-07 | CC |
| GO:0044436 | Obsolete thylakoid part | 1.42E-06 | CC |
| GO:0048046 | Apoplast | 8.24E-06 | CC |
| GO:0030312 | External encapsulating structure | 3.19E-05 | CC |
| GO:0005576 | Extracellular region | 3.44E-05 | CC |
| GO:0009266 | Response to temperature stimulus | 1.38E-04 | BP |
| GO:0005618 | Cell wall | 3.58E-04 | CC |
| GO:0009505 | Plant-type cell wall | 3.58E-04 | CC |
| GO:0009941 | Chloroplast envelope | 3.58E-04 | CC |
| GO:0009408 | Response to heat | 4.06E-04 | BP |
| GO:0009526 | Plastid envelope | 5.70E-04 | CC |
| GO:0009414 | Response to water deprivation | 0.002018 | BP |
| GO:0009415 | Response to water | 0.002018 | BP |
| GO:0009644 | Response to high light intensity | 0.002018 | BP |
| GO:0009409 | Response to cold | 0.014081 | BP |
| GO:0022625 | Cytosolic large ribosomal subunit | 0.014275 | CC |
| GO:0010287 | Plastoglobule | 0.014941 | CC |
| GO:0009570 | Chloroplast stroma | 0.018685 | CC |
| GO:0009532 | Plastid stroma | 0.019715 | CC |
| GO:0015934 | Large ribosomal subunit | 0.022132 | CC |
| GO:0009765 | Photosynthesis, light harvesting | 0.025471 | BP |
| GO:0090376 | Seed trichome differentiation | 0.026689 | BP |
| GO:0090378 | Seed trichome elongation | 0.026689 | BP |
| GO:0034605 | Cellular response to heat | 0.026689 | BP |
| GO:0016168 | Chlorophyll binding | 0.033641 | MF |
| GO:0046906 | Tetrapyrrole binding | 0.033641 | MF |
| GO:0098807 | Chloroplast thylakoid membrane protein complex | 0.034039 | CC |
| GO:0015979 | Photosynthesis | 0.03525 | BP |
| GO:0006869 | Lipid transport | 0.03525 | BP |
| GO:0009642 | Response to light intensity | 0.040788 | BP |
| GO:0044391 | Ribosomal subunit | 0.041104 | CC |
| GO:0000326 | Protein storage vacuole | 0.043414 | CC |

**Table S16** Gene Ontology (GO) enrichment analysis of differentially expressed syntenic gene pairs between *Eremogone kansuensis* and *E. juncea* var. *juncea* in root (significantly enriched terms, FDR < 0.05).

| **ID** | **Term Name** | **p. adjust** | **Category** |
| --- | --- | --- | --- |
| GO:0009505 | Plant-type cell wall | 1.79E-04 | CC |
| GO:0030312 | External encapsulating structure | 1.79E-04 | CC |
| GO:0005618 | Cell wall | 3.40E-04 | CC |
| GO:0009414 | Response to water deprivation | 0.00829 | BP |
| GO:0009415 | Response to water | 0.00829 | BP |
| GO:0009266 | Response to temperature stimulus | 0.00829 | BP |
| GO:0042493 | Response to xenobiotic stimulus | 0.009942 | BP |
| GO:0006080 | Substituted mannan metabolic process | 0.009942 | BP |
| GO:0010392 | Galactoglucomannan metabolic process | 0.009942 | BP |
| GO:0051069 | Galactomannan metabolic process | 0.009942 | BP |
| GO:0051070 | Galactomannan biosynthetic process | 0.009942 | BP |
| GO:0010200 | Response to chitin | 0.009942 | BP |
| GO:0019359 | Nicotinamide nucleotide biosynthetic process | 0.021331 | BP |
| GO:0019363 | Pyridine nucleotide biosynthetic process | 0.022478 | BP |
| GO:0043169 | Cation binding | 0.02296 | MF |
| GO:0072525 | Pyridine-containing compound biosynthetic process | 0.027318 | BP |
| GO:0009409 | Response to cold | 0.027524 | BP |
| GO:0098542 | Defense response to other organism | 0.031823 | BP |
| GO:0009737 | Response to abscisic acid | 0.031823 | BP |
| GO:0006096 | Glycolytic process | 0.031823 | BP |
| GO:0006757 | Obsolete ATP generation from ADP | 0.031823 | BP |
| GO:0006090 | Pyruvate metabolic process | 0.031823 | BP |
| GO:0042866 | Pyruvate biosynthetic process | 0.031823 | BP |
| GO:0046031 | ADP metabolic process | 0.031823 | BP |
| GO:0009135 | Purine nucleoside diphosphate metabolic process | 0.031823 | BP |
| GO:0009179 | Purine ribonucleoside diphosphate metabolic process | 0.031823 | BP |
| GO:0009185 | Ribonucleoside diphosphate metabolic process | 0.031823 | BP |
| GO:0010191 | Obsolete mucilage metabolic process | 0.031823 | BP |
| GO:0072330 | Monocarboxylic acid biosynthetic process | 0.032008 | BP |
| GO:0009408 | Response to heat | 0.032497 | BP |
| GO:0097305 | Response to alcohol | 0.033484 | BP |
| GO:0009644 | Response to high light intensity | 0.042356 | BP |
| GO:0050832 | Defense response to fungus | 0.042356 | BP |
| GO:0009620 | Response to fungus | 0.047967 | BP |
| GO:0006165 | Obsolete nucleoside diphosphate phosphorylation | 0.048529 | BP |

**Table S17** Gene Ontology (GO) enrichment analysis of differentially expressed syntenic gene pairs between *Eremogone kansuensis* and *E. juncea* var. *juncea* in stem (significantly enriched terms, FDR < 0.05).

| **ID** | **Term Name** | **p. adjust** | **Category** |
| --- | --- | --- | --- |
| GO:0009534 | Chloroplast thylakoid | 5.20E-17 | CC |
| GO:0031976 | Plastid thylakoid | 5.20E-17 | CC |
| GO:0034357 | Photosynthetic membrane | 1.53E-16 | CC |
| GO:0055035 | Plastid thylakoid membrane | 1.53E-16 | CC |
| GO:0009579 | Thylakoid | 4.11E-16 | CC |
| GO:0009535 | Chloroplast thylakoid membrane | 4.11E-16 | CC |
| GO:0042651 | Thylakoid membrane | 5.30E-16 | CC |
| GO:0009768 | Photosynthesis, light harvesting in photosystem I | 1.43E-11 | BP |
| GO:0031409 | Pigment binding | 1.05E-10 | MF |
| GO:0010287 | Plastoglobule | 1.20E-10 | CC |
| GO:0016168 | Chlorophyll binding | 1.13E-09 | MF |
| GO:0046906 | Tetrapyrrole binding | 4.34E-08 | MF |
| GO:0019904 | Protein domain specific binding | 1.21E-07 | MF |
| GO:0015979 | Photosynthesis | 1.60E-07 | BP |
| GO:0009765 | Photosynthesis, light harvesting | 1.60E-07 | BP |
| GO:0009414 | Response to water deprivation | 1.41E-06 | BP |
| GO:0009415 | Response to water | 1.53E-06 | BP |
| GO:0009644 | Response to high light intensity | 1.60E-06 | BP |
| GO:0009409 | Response to cold | 1.60E-06 | BP |
| GO:0009526 | Plastid envelope | 1.93E-06 | CC |
| GO:0009941 | Chloroplast envelope | 2.19E-06 | CC |
| GO:0009505 | Plant-type cell wall | 7.07E-06 | CC |
| GO:0009570 | Chloroplast stroma | 8.25E-06 | CC |
| GO:0048046 | Apoplast | 9.37E-06 | CC |
| GO:0009532 | Plastid stroma | 9.37E-06 | CC |
| GO:0019684 | Photosynthesis, light reaction | 1.10E-05 | BP |
| GO:0009642 | Response to light intensity | 1.23E-05 | BP |
| GO:0009266 | Response to temperature stimulus | 1.87E-05 | BP |
| GO:0080167 | Response to karrikin | 1.87E-05 | BP |
| GO:0009645 | Response to low light intensity stimulus | 2.58E-04 | BP |
| GO:0009611 | Response to wounding | 2.94E-04 | BP |
| GO:0005576 | Extracellular region | 2.97E-04 | CC |
| GO:0030312 | External encapsulating structure | 3.76E-04 | CC |
| GO:0098807 | Chloroplast thylakoid membrane protein complex | 7.97E-04 | CC |
| GO:0005618 | Cell wall | 8.26E-04 | CC |
| GO:0006091 | Generation of precursor metabolites and energy | 8.80E-04 | BP |
| GO:0005509 | Calcium ion binding | 8.94E-04 | MF |
| GO:0016798 | Hydrolase activity, acting on glycosyl bonds | 0.001042 | MF |

**Table S17 (continued)** Gene Ontology (GO) enrichment analysis of differentially expressed syntenic gene pairs between *Eremogone kansuensis* and *E. juncea* var. *juncea* in stem (significantly enriched terms, FDR < 0.05).

| **ID** | **Term Name** | **p. adjust** | **Category** |
| --- | --- | --- | --- |
| GO:0000326 | Protein storage vacuole | 0.001494 | CC |
| GO:0009617 | Response to bacterium | 0.003195 | BP |
| GO:0098542 | Defense response to other organism | 0.003505 | BP |
| GO:0002213 | Defense response to insect | 0.003505 | BP |
| GO:0000322 | Storage vacuole | 0.012187 | CC |
| GO:0097305 | Response to alcohol | 0.012511 | BP |
| GO:0009737 | Response to abscisic acid | 0.017556 | BP |
| GO:0009753 | Response to jasmonic acid | 0.017556 | BP |
| GO:0048037 | Obsolete cofactor binding | 0.021746 | MF |
| GO:0006979 | Response to oxidative stress | 0.02221 | BP |
| GO:0042742 | Defense response to bacterium | 0.02221 | BP |
| GO:0010200 | Response to chitin | 0.02221 | BP |
| GO:0022626 | Cytosolic ribosome | 0.023653 | CC |
| GO:0010218 | Response to far red light | 0.024374 | BP |
| GO:0005840 | Ribosome | 0.026562 | CC |
| GO:0004791 | Thioredoxin-disulfide reductase (NADPH) activity | 0.030471 | MF |
| GO:0005372 | Water transmembrane transporter activity | 0.030471 | MF |
| GO:0015250 | Water channel activity | 0.030471 | MF |
| GO:0000786 | Nucleosome | 0.030508 | CC |
| GO:0031225 | Obsolete anchored component of membrane | 0.031252 | CC |
| GO:0044815 | Obsolete DNA packaging complex | 0.040557 | CC |
| GO:0046658 | Obsolete anchored component of plasma membrane | 0.046843 | CC |

**Table S18** Gene Ontology (GO) enrichment analysis of differentially expressed syntenic gene pairs between *Eremogone kansuensis* and *E. juncea* var. *juncea* in flower (significantly enriched terms, FDR < 0.05).

| **ID** | **Term Name** | **p. adjust** | **Category** |
| --- | --- | --- | --- |
| GO:0009534 | Chloroplast thylakoid | 6.02E-08 | CC |
| GO:0031976 | Plastid thylakoid | 6.02E-08 | CC |
| GO:0009535 | Chloroplast thylakoid membrane | 2.89E-07 | CC |
| GO:0055035 | Plastid thylakoid membrane | 2.89E-07 | CC |
| GO:0042651 | Thylakoid membrane | 4.12E-07 | CC |
| GO:0034357 | Photosynthetic membrane | 4.12E-07 | CC |
| GO:0009579 | Thylakoid | 4.12E-07 | CC |
| GO:0048046 | Apoplast | 8.24E-06 | CC |
| GO:0030312 | External encapsulating structure | 3.19E-05 | CC |
| GO:0005576 | Extracellular region | 3.44E-05 | CC |
| GO:0009266 | Response to temperature stimulus | 1.38E-04 | BP |
| GO:0005618 | Cell wall | 3.58E-04 | CC |
| GO:0009505 | Plant-type cell wall | 3.58E-04 | CC |
| GO:0009941 | Chloroplast envelope | 3.58E-04 | CC |
| GO:0009408 | Response to heat | 4.06E-04 | BP |
| GO:0009526 | Plastid envelope | 5.70E-04 | CC |
| GO:0009414 | Response to water deprivation | 0.002018 | BP |
| GO:0009415 | Response to water | 0.002018 | BP |
| GO:0009644 | Response to high light intensity | 0.002018 | BP |
| GO:0009409 | Response to cold | 0.014081 | BP |
| GO:0022625 | Cytosolic large ribosomal subunit | 0.014275 | CC |
| GO:0010287 | Plastoglobule | 0.014941 | CC |
| GO:0009570 | Chloroplast stroma | 0.018685 | CC |
| GO:0009532 | Plastid stroma | 0.019715 | CC |
| GO:0015934 | Large ribosomal subunit | 0.022132 | CC |
| GO:0009765 | Photosynthesis, light harvesting | 0.025471 | BP |
| GO:0090376 | Seed trichome differentiation | 0.026689 | BP |
| GO:0090378 | Seed trichome elongation | 0.026689 | BP |
| GO:0034605 | Cellular response to heat | 0.026689 | BP |
| GO:0016168 | Chlorophyll binding | 0.033641 | MF |
| GO:0046906 | Tetrapyrrole binding | 0.033641 | MF |
| GO:0098807 | Chloroplast thylakoid membrane protein complex | 0.034039 | CC |
| GO:0015979 | Photosynthesis | 0.03525 | BP |
| GO:0006869 | Lipid transport | 0.03525 | BP |
| GO:0009642 | Response to light intensity | 0.040788 | BP |
| GO:0044391 | Ribosomal subunit | 0.041104 | CC |
| GO:0000326 | Protein storage vacuole | 0.043414 | CC |

**Table S19** KEGG pathway enrichment analysis of differentially expressed syntenic gene pairs between *Eremogone kansuensis* and *E. juncea* var. *juncea*.

| **Term ID** | **Term Name** | **Category** | **p. adjust** |
| --- | --- | --- | --- |
| map00195 | Photosynthesis | KEGG | 1.40E-16 |
| map00196 | Photosynthesis - antenna proteins | KEGG | 1.07E-11 |
| map00710 | Carbon fixation by Calvin cycle | KEGG | 6.16E-05 |
| map04626 | Plant-pathogen interaction | KEGG | 0.002684 |
| map00630 | Glyoxylate and dicarboxylate metabolism | KEGG | 0.004334 |
| map00940 | Phenylpropanoid biosynthesis | KEGG | 0.006649 |
| map01200 | Carbon metabolism | KEGG | 0.007696 |
| map00941 | Flavonoid biosynthesis | KEGG | 0.040766 |

**Materials and Methods**

**Plant materials**

Fresh young leaves of *Eremogone kansuensis* were collected in August 2024 from Xianmi National Park, Menyuan Hui Autonomous County, Haibei Tibetan Autonomous Prefecture, Qinghai Province, China (37°20′46.78728″ N, 101°23′10.71456″ E; 3,983.9 m). The sampled individuals were naturally growing on high-elevation alpine scree slopes in the subnival belt, characterized by loose rocky debris and sparse vegetation. Fresh young leaves of its low-altitude relative *Eremogone juncea* var. *juncea* were collected in September 2024 from Wubao’an Mountain, Zoucheng City, Shandong Province, China (31°08′21.35″ N, 110°25′48.02″ E; 445 m). The sampled individuals were collected from rocky hillside grassland habitats at low elevation. Leaf samples from both species were used for genome sequencing. For transcriptome analyses, root, stem, leaf, and flower tissues were sampled from naturally growing individuals of each species, with three independent biological replicates per tissue. All plant materials were immediately frozen in liquid nitrogen after collection and stored at −80 °C until DNA or RNA extraction.

**Library construction and sequencing**

SMRTbell libraries were constructed following the manufacturer’s protocol (Pacific Biosciences, CA, USA) using a 15 kb size-selection strategy. Approximately 15 μg of high-molecular-weight genomic DNA was sheared using g-TUBEs (Covaris, USA) to obtain target fragment sizes. After single-strand overhang removal, DNA fragments were subjected to damage repair, end repair, and A-tailing, followed by ligation to SMRTbell hairpin adapters. The libraries were treated with the SMRTbell Enzyme Cleanup Kit and purified using AMPure PB beads. Size selection was performed on a BluePippin system (Sage Science, USA), followed by additional purification with AMPure PB beads. Fragment size distributions were assessed using an Agilent 2100 Bioanalyzer (Agilent Technologies, USA). Sequencing was carried out on the PacBio Sequel II platform using Sequencing Primer V2 and Sequel II Binding Kit 2.1. For Hi-C library construction, fresh leaf tissue was cross-linked with 1% formaldehyde and digested using the DpnII restriction enzyme. Proximity-ligated DNA fragments were processed following standard Hi-C library preparation procedures and sequenced on an Illumina HiSeq 4000 platform.

**Genome size estimation and assembly**

*K*-mer depth distributions were calculated from PacBio HiFi reads using Jellyfish (v.2.10) (Marçais and Kingsford, 2011), with *K*-mer sizes of 31. The resulting *K*-mer spectra were then modeled using GenomeScope v.1.0.0 (Vurture et al., 2017), which enabled the prediction of genomic characteristics, including genome size and heterozygosity. High-accuracy continuous long-read (CCS) data were first generated and used for contig-level genome assembly with hifiasm (version 0.19.5) (Cheng et al., 2021). To detect and remove uncollapsed duplications, a subprogram of Purge Haplotigs (v.1.1.2) (Roach et al., 2018) together with purge_dup (v.1.2.5) (Guan et al., 2020)was applied. Genome assembly completeness was subsequently assessed using BUSCO (Benchmarking Universal Single-Copy Orthologs, v5.4.3) (Simão et al., 2015), with the lineage-specific database *eudicots_odb10* employed for accurate evaluation of genome integrity. Hi-C data were then incorporated for chromosome-scale scaffolding. Raw Hi-C reads generated using MboI digestion were first processed with HiC-Pro (v.3.1.0) (Servant et al., 2015) to obtain clean Hi-C data. These filtered reads were aligned to the assembled genome using Juicer (v.1.6) (Durand et al., 2016). Chromosome-level scaffolding was performed with HapHic (v.1.0.7) (Zeng et al., 2024), which clustered, ordered, and oriented contigs based on Hi-C interaction information. Finally, Hi-C contact matrices were visualized in Juicebox (v.1.11.08), resulting in the anchoring of assembled sequences into 11 super-assemblies corresponding to 11 pseudo-chromosomes for each species.

**Repeat element identification and gene prediction**

RepeatMasker (Tarailo-Graovac and Chen, 2009) and RepeatProteinMasker (Tarailo-Graovac and Chen, 2009) were used to identify repetitive elements based on homology alignments between the *Eremogone kansuensis* and *Eremogone juncea* var. *juncea* genome sequences and Repbase (v.16.10) (Bao et al., 2015). We then applied a de novo strategy to increase the sensitivity of repeat detection. Briefly, RepeatModeler and LTR_Finder (v1.06) (Xu and Wang, 2007) were used to construct a repeat library, and RepeatMasker (Tarailo-Graovac and Chen, 2009) was subsequently applied to generate de novo repeat annotations. We used three complementary strategies to predict and annotate genes in the *E. kansuensis* and *E. juncea* genomes, including ab initio, homology-based, and transcriptome-based methods. For ab initio prediction, Augustus (v.3.2.3) (Stanke and Morgenstern, 2005), Helixer (v.0.3.6) (Holst et al., 2025), and Eviann (v.2.0.4) (Zimin et al., 2025) were employed. For homology-based prediction, GeMoMa (v.1.7.1) (Keilwagen et al., 2019) was applied using protein sequences from *Gymnocarpos paniculata* (Li et al., 2022), *Gymnocarpos vaccaria*, *Dianthus caryophyllus* (Lan et al., 2024), *Saponaria officinalis* (Jo et al., 2025), *Silene latifolia*, *Silene dioica*, *Silene vulgaris* (Akagi et al., 2025), *Silene conica*, and *Heliosperma pusillum* (Szukala et al., 2023). For transcriptome-based prediction, RNA-seq reads were aligned to the genome using HISAT2 (v.2.2.1) (Kim et al., 2019) and assembled into gene models using TransDecoder (v5.1.0, https://github.com/TransDecoder/TransDecoder). Finally, all gene models from different approaches were integrated into a non-redundant gene set using Evidence Modeler (EVM, v.1.11) (Haas et al., 2008), and annotation completeness was evaluated with BUSCO (version 5.4.3). Eggnog (Cantalapiedra et al., 2021)and iTAK (v2.0.2) (Zheng et al., 2016) were used to functional annotation of gene models.

**Genome evolution and expansion/contraction of gene families**

To investigate genome evolution and gene family dynamics in Caryophyllaceae, 12 representative species from seven genera were selected, with *Vitis vinifera* used as an outgroup. The sampled taxa included *Gymnocarpos przewalskii* (*Gymnocarpos*) (Fu et al., 2024), *Spergularia bocconei* (*Spergularia*) , *Gypsophila paniculata* (Li et al., 2022) and *Gypsophila vaccaria* (*Gypsophila*) (Zhang et al., 2025), *Dianthus caryophyllus* (*Dianthus*) (Lan et al., 2024), *Saponaria officinalis* (*Saponaria*) (Jo et al., 2025), *Eremogone kansuensis* and *Eremogone juncea* var. *juncea* (*Eremogone*), and four *Silene* species (*Silene latifolia*, *Silene dioica*, *Silene conica*, and *Silene vulgaris*) (Akagi et al., 2025; Fields et al., 2023).

Orthologous gene families among all sampled species were identified using OrthoFinder v2.5.5 (Emms and Kelly, 2019) with default parameters, and single-copy orthologous genes shared among all species were extracted for phylogenetic reconstruction. For each orthologous group, protein sequences were aligned using MAFFT (Katoh et al., 2002) with default settings, and the resulting protein alignments were converted into corresponding codon alignments using PAL2NAL (Suyama et al., 2006), ensuring that nucleotide alignments were guided by the protein alignments to preserve codon structure. All codon alignments of single-copy orthologs were concatenated into a single supermatrix using in-house scripts and used for subsequent phylogenetic inference.

Phylogenetic reconstruction was conducted using IQ-TREE v2.4.0 (Nguyen et al., 2015) with the parameters “-bb 1000 -m MFP -nt AUTO”. The best-fit nucleotide substitution model was automatically selected using ModelFinder implemented in IQ-TREE, and according to the Bayesian Information Criterion (BIC), the optimal model selected for the concatenated dataset was GTR+F+R3. Branch support values were estimated using 1000 ultrafast bootstrap replicates.

Divergence time estimation was performed using MCMCtree implemented in the PAML package (Yang, 2007). The analysis was conducted using the approximate likelihood method (usedata = 2) under a correlated relaxed molecular clock model. The substitution model was set to the GTR model (model = 7), with among-site rate variation modeled using a gamma distribution (alpha = 0.5, ncatG = 5). Ambiguous sites were retained in the alignment (cleandata = 0). The following prior settings were applied: BDparas = 1 1 0 for the birth–death process; kappa_gamma = 6 2; alpha_gamma = 1 1; rgene_gamma = 1 4.05616; and sigma2_gamma = 1 4.5. Markov chain Monte Carlo (MCMC) sampling was performed with a burn-in of 500,000 iterations, followed by 20,000 samples collected every 5,000 iterations (burnin = 500000, sampfreq = 5000, nsample = 20000). Convergence to the stationary distribution was assessed using Tracer v1.7.2 (Rambaut et al., 2018) by examining effective sample size (ESS) values for all estimated parameters.

Two calibration constraints were applied, including the divergence between Caryophyllaceae and Vitaceae, constrained to 111.4-123.9 million years ago (Mya) based on estimates obtained from the TimeTree database (<https://timetree.org/>) (Hedges et al., 2006), and the divergence between *Gypsophila vaccaria* and *Spergularia bocconei*, constrained to 34.2–56.1 Mya based on published divergence time estimates.

The final phylogenetic tree was drawn using the online tool Chiplot (Xie et al., 2023) (<https://www.chiplot.online/>) and subsequently refined in Adobe Illustrator (version 26.0.1).

Gene family expansion and contraction analyses were conducted using CAFÉ v5 (Mendes et al., 2021), based on the inferred phylogenetic tree and the gene family clustering results obtained from OrthoFinder.

**Analysis of whole-genome duplication and subgenome identification**

Whole-genome duplication (WGD) events and subgenome identification were analyzed using the Whole-Genome Duplication Integrated (WGDI) pipeline (Sun et al., 2022). A total of 12 Caryophyllaceae species representing seven genera were included in the analysis. *Vitis vinifera*, which retains a genome structure derived from the ancestral γ whole-genome triplication shared by core eudicots, was used in genome-wide synteny dot-plot comparisons to evaluate duplication depth relative to the ancestral γ background.

Syntenic depth relationships between *Vitis vinifera* and each Caryophyllaceae genome were examined to infer genome multiplication patterns. A 1:1 relationship indicates retention of only the γ-derived structure, whereas 1:2 and 1:3 relationships indicate one additional whole-genome duplication or triplication beyond the ancestral γ event, respectively.

To infer genome-wide duplication signals, synonymous substitution rate (*Ks*) distributions were calculated using WGDI with the “-ks”, “-kp”, and “-pf” parameters, which compute *Ks* values for collinear gene pairs, plot *Ks* distributions, and fit *Ks* peaks, respectively.

Subgenome identification was subsequently performed by mapping ancestral genomic blocks onto each genome using the “-km” option in WGDI. Duplicated genomic regions were assigned into distinct subgenome sets, and collinear genes corresponding to each subgenome were extracted using the “-pc” and “-a” parameters.

To investigate phylogenetic relationships among collinear gene sets, protein sequences from each collinear gene set were aligned using MAFFT (Katoh et al., 2002), followed by trimming using trimAl to remove poorly aligned regions (minimum overlap = 4; delete_detail = true). Gene trees were then constructed for each collinear gene set using IQ-TREE under the ModelFinder option (MFP) to select the best-fit substitution model, and branch support values were estimated using the ultrafast bootstrap method with 1,000 replicates (-bb 1000). Finally, all remaining gene trees were integrated using ASTRAL (v5.7.8) to infer species-level relationships from individual gene trees. (Zhang et al., 2018).

To assess whether genome triplication events were lineage-specific or shared across species, pairwise comparisons were conducted among the 11 Caryophyllaceae species that exhibited a 1:3 syntenic relationship relative to *Vitis vinifera*, indicating the presence of an additional genome triplication event following the ancestral γ event (excluding *Spergularia bocconei*). In total, 55 pairwise combinations were generated. For each pairwise comparison, collinear gene sets containing at least two homologous genes from each species were extracted, and gene trees were reconstructed as described above. The resulting tree topologies were classified according to whether homologous genes from the two species were intermingled within the same duplication-derived clades (indicating shared duplication events) or grouped into separate species-specific clades (indicating lineage-specific duplication patterns; e.g., mixed clustering patterns such as ((A1, B1), (A2, B2), (A3, B3)) versus species-specific clustering such as ((A1, A2, A3), (B1, B2, B3))). The proportions of gene trees showing intermingled topologies were quantified across all pairwise comparisons to evaluate whether genome triplication signals were shared between species.

**Ancestral Caryophyllaceae karyotype construction and karyotype evolution**

Homologous gene arrangements across chromosomes were examined using pairwise collinearity dot‐plot analyses to identify orthologous and paralogous relationships and to detect signals of chromosomal fusion and rearrangement events. Pairwise synteny analyses were performed among all Caryophyllaceae species included in this study using the WGDI pipeline (<https://github.com/SunPengChuan/wgdi>) (Wang et al., 2022; Sun et al., 2022, 2024), and collinear gene pairs were identified based on conserved gene order. Based on genome-wide collinearity patterns, conserved chromosome-like synteny blocks (CLSBs) shared across species were identified as candidate ancestral chromosomal segments. These CLSBs were iteratively integrated following the WGDI workflow to reconstruct the ancestral Caryophyllaceae karyotype (ACK). In each iteration, conserved CLSBs showing continuous collinearity across species were incorporated into provisional ancestral chromosomal segments, and the remaining regions were further examined in subsequent rounds until all major ancestral segments were recovered. During this process, several chromosomal regions exhibiting gapless or nearly continuous collinearity across multiple species were identified. In particular, chromosomes Sbo01, Sbo08, Sbo17, Sbo12, Sbo05, Sbo13, and Sbo10 from *Spergularia bocconei* showed long and continuous collinear relationships with corresponding regions in multiple other species, indicating strong conservation of ancestral chromosomal structure. For ancestral chromosomes that were not fully represented by a single extant chromosome, additional CLSBs from other species were incorporated iteratively. For example, segments from *Gypsophila paniculata* (Gpa02), *Spergularia bocconei* (Sbo02), and *Gypsophila vaccaria* (Gva14) were integrated to reconstruct ACK7, whereas segments from *Gymnocarpos przewalskii* (Gpr01) together with Sbo14 contributed to the reconstruction of ACK8. Through iterative integration of conserved CLSBs across species, nine ancestral chromosomal segments were ultimately recovered, resulting in the reconstruction of an ancestral Caryophyllaceae karyotype (ACK) consisting of nine chromosomes (*n* = 9). Chromosomal fusion events were identified by performing collinearity dot-plot analyses between the reconstructed ancestral Caryophyllaceae karyotype (ACK) and extant genomes. Fusion events were inferred when genomic regions corresponding to two ancestral protochromosomes were detected within a single extant chromosome in the dot-plot patterns. Shared fusion events were determined when the same combination of ancestral protochromosomes and similar fusion boundaries were consistently observed across multiple species within a lineage. Representative fusion signals were confirmed through visual inspection of chromosome-level dot plots.

**RNA sequencing**

Total RNA was extracted from each tissue using the RNeasy plant RNA extraction kit. RNA-seq libraries were constructed using standard protocols and sequenced on DNBSEQ-T7 sequencing platform in paired-end mode. In total, 173.2Gb of paired-end Illumina RNA-seq data were generated across all samples. Raw reads were subjected to stringent quality control to remove adapter sequences, reads containing excessive ambiguous bases (N > 8), low-complexity reads, and low-quality reads (more than 50% of bases with a Phred quality score < 5). The resulting clean reads were retained for subsequent transcriptome assembly and gene expression analyses.

**Gene retention and expression dominance**

Gene retention after the shared α WGT was inferred relative to the ACK by comparing each genome to the ACK separately. For each ACK gene, we counted the number of syntenic orthologs recovered in each species and assigned it to a corresponding copy-number retention category. We then constructed a species-by-ACK copy-number matrix and calculated among-species dissimilarities using the Manhattan distance implemented in the R package vegan. These distances were visualized by PCoA, and the association between retention-pattern distances and species’ tribes was evaluated using PERMANOVA (999 permutations), reporting *P*-value and R^2^.

**Cross-species transcriptomic analysis**

Clean RNA-seq reads were independently aligned to the corresponding reference genomes of *Eremogone kansuensis* and *E. juncea* var. *juncea* using HISAT2 (Kim et al., 2019) with default parameters. Transcript assemblies and gene-level expression estimates for each sample were generated using StringTie based on the genome-guided alignments. Raw read count matrices were subsequently extracted using the prepDE.py script. Syntenic gene pairs between *E. kansuensis* and *E. juncea* var. *juncea* were identified using WGDI, and all downstream comparative transcriptomic analyses were restricted to these syntenic gene pairs. However, even when conservative one-to-one syntenic orthologs are used, cross-species RNA-seq comparisons can still be affected by differences in gene length between species as well as variation in sequencing depth across samples. To ensure the reliability and comparability of expression estimates, we adopted a previously described normalization strategy based on GeTMM (gene-length corrected trimmed mean of M-values) (Smid et al., 2018). GeTMM integrates gene length normalization with the trimmed mean of M-values (TMM) normalization procedure implemented in edgeR, allowing expression levels of synteny genes to be directly comparable between species. Following the previously described method (Ma et al., 2023), each species was treated as a separate group, and TMM normalization factors were calculated to adjust for differences in library size. Gene expression values were then normalized for both gene length and sequencing depth to obtain GeTMM values, which were used for all subsequent cross-species transcriptomic analyses.

Principal component analysis (PCA) was performed on the GeTMM-normalized expression matrix using the “prcomp” function implemented in the R package stats. Pairwise Pearson correlation coefficients between samples were calculated using the “cor” function in R. Hierarchical clustering analysis was conducted based on the same normalized dataset using the “hcluster” function in the amap package Differential expression analysis of synteny gene pairs was performed by edgeR using the Cox–Reid profile-adjusted likelihood (CR) method, which implemented in the “estimateDisp” function. Differentially expressed genes (DEGs) were defined based on a threshold of |logFC| > 1 and a Benjamini-Hochberg false discovery rate (FDR)-adjusted *P* value < 0.05.

To further investigate the functional roles of differentially expressed syntenic gene pairs, we identified their corresponding orthologs in the model plant *Arabidopsis thaliana*. Orthology assignment was performed based on sequence similarity (BLAST identity > 0.4) and the presence of fully conserved Pfam domain architectures. Functional annotation of the *A. thaliana* orthologs was conducted using the stress-related gene annotations curated in the Stress Responsive Transcription Factor Database (STIFDB2) (Naika et al., 2013), with a focus on genes implicated in cold stress, oxidative stress, and UV-B stress responses. In addition, stress-responsive interaction networks for the differentially expressed syntenic gene pairs were constructed using the Stress Knowledge Map (SKM; https://skm.nib.si/). Interaction networks were assembled using interaction evidence ranging from Rank 0 to Rank 2, including manually curated interactions, literature-curated interactions, and interactions identified exclusively through high-throughput experimental approaches.

**Enrichment and statistical analysis**

GO and KEGG enrichment analyses were performed using the enricher function in the R package clusterProfiler. *P* values were adjusted for multiple testing using the Benjamini–Hochberg (BH) method. GO terms and KEGG pathways with adjusted *P* values (padj) < 0.05 were considered significantly enriched, and those with padj < 0.01 were selected for visualization. Interaction networks were visualized using Cytoscape. Statistical analyses were conducted in R (v4.4.1; https://www.r-project.org/), and results were visualized using the ggplot2 package (v3.5.1; https://ggplot2.tidyverse.org/). Group differences were assessed using the Wilcoxon rank-sum test (two-sided, confidence level = 0.95). Differences in categorical variables between groups were evaluated using the chi-square test.

**References**

Akagi, T., Fujita, N., Shirasawa, K., et al., 2025. Rapid and dynamic evolution of a giant Y chromosome in *Silene latifolia*. Science 387, 637–643. https://doi.org/10.1126/science.adk9074.

Bao, W., Kojima, K.K., Kohany, O., 2015. Repbase Update, a database of repetitive elements in eukaryotic genomes. Mob. DNA 6, 11. https://doi.org/10.1186/s13100-015-0041-9.

Cantalapiedra, C.P., Hernández-Plaza, A., Letunic, I., et al., 2021. eggNOG-mapper v2: functional annotation, orthology assignments, and domain prediction at the metagenomic scale. Mol. Biol. Evol. 38, 5825–5829. https://doi.org/10.1093/molbev/msab293.

Cheng, H., Concepcion, G.T., Feng, X., et al., 2021. Haplotype-resolved de novo assembly using phased assembly graphs with hifiasm. Nat. Methods 18, 170–175. https://doi.org/10.1038/s41592-020-01056-5.

Durand, N.C., Shamim, M.S., Machol, I., et al., 2016. Juicer provides a one-click system for analyzing loop-resolution Hi-C experiments. Cell Syst. 3, 95–98. https://doi.org/10.1016/j.cels.2016.07.002.

Emms, D.M., Kelly, S., 2019. OrthoFinder: phylogenetic orthology inference for comparative genomics. Genome Biol. 20, 238. https://doi.org/10.1186/s13059-019-1832-y.

Fields, P.D., Weber, M.M., Waneka, G., et al., 2023. Chromosome-level genome assembly for the angiosperm *Silene conica*. Genome Biol. Evol. 15, evad192. https://doi.org/10.1093/gbe/evad192.

Fu, R., Zhu, Y., Liu, Y., et al., 2024. Shared xerophytic genes and their re‐use in local adaptation to aridity in the desert plant *Gymnocarpos przewalskii*. Mol. Ecol. 33, e17380. https://doi.org/10.1111/mec.17380.

Guan, D., McCarthy, S.A., Wood, J., et al., 2020. Identifying and removing haplotypic duplication in primary genome assemblies. Bioinformatics 36, 2896–2898. https://doi.org/10.1093/bioinformatics/btaa025.

Haas, B.J., Salzberg, S.L., Zhu, W., et al., 2008. Automated eukaryotic gene structure annotation using EVidenceModeler and the program to assemble spliced alignments. Genome Biol. 9, R7. https://doi.org/10.1186/gb-2008-9-1-r7.

Hedges, S.B., Dudley, J., Kumar, S., 2006. TimeTree: a public knowledge-base of divergence times among organisms. Bioinformatics 22, 2971–2972. https://doi.org/10.1093/bioinformatics/btl505.

Holst, F., Bolger, A.M., Kindel, F., et al., 2025. Helixer: ab initio prediction of primary eukaryotic gene models combining deep learning and a hidden Markov model. Nat. Methods. https://doi.org/10.1038/s41592-025-02939-1.

Jo, S., El-Demerdash, A., Owen, C., et al., 2025. Unlocking saponin biosynthesis in soapwort. Nat. Chem. Biol. 21, 215–226. https://doi.org/10.1038/s41589-024-01681-7.

Katoh, K., Misawa, K., Kuma, K., et al., 2002. MAFFT: a novel method for rapid multiple sequence alignment based on fast Fourier transform. Nucleic Acids Res. 30, 3059–3066. https://doi.org/10.1093/nar/gkf436.

Keilwagen, J., Hartung, F., Grau, J., 2019. GeMoMa: homology-based gene prediction utilizing intron position conservation and RNA-seq data. Methods Mol. Biol. Clifton NJ 1962, 161–177. https://doi.org/10.1007/978-1-4939-9173-0_9.

Kim, D., Paggi, J.M., Park, C., et al., 2019. Graph-based genome alignment and genotyping with HISAT2 and HISAT-genotype. Nat. Biotechnol. 37, 907–915. https://doi.org/10.1038/s41587-019-0201-4.

Lan, L., Leng, L., Liu, W., et al., 2024. The haplotype-resolved telomere-to-telomere carnation (*Dianthus caryophyllus*) genome reveals the correlation between genome architecture and gene expression. Hortic. Res. 11, uhad244. https://doi.org/10.1093/hr/uhad244.

Li, F., Gao, Y., Jin, C., et al., 2022. The chromosome-level genome of *Gypsophila paniculata* reveals the molecular mechanism of floral development and ethylene insensitivity. Hortic. Res. 9, uhac176. https://doi.org/10.1093/hr/uhac176.

Ma, J., Zhang, L., Shen, F., et al., 2023. Gene expressions between obligate bamboo-eating pandas and non-herbivorous mammals reveal converged specialized bamboo diet adaptation. BMC Genomics 24, 23. https://doi.org/10.1186/s12864-023-09111-z.

Marçais, G., Kingsford, C., 2011. A fast, lock-free approach for efficient parallel counting of occurrences of k-mers. Bioinformatics 27, 764–770. https://doi.org/10.1093/bioinformatics/btr011.

Mendes, F.K., Vanderpool, D., Fulton, B., et al., 2021. CAFE 5 models variation in evolutionary rates among gene families. Bioinformatics 36, 5516–5518. https://doi.org/10.1093/bioinformatics/btaa1022.

Naika, M., Shameer, K., Mathew, O.K., et al., 2013. STIFDB2: an updated version of plant stress-responsive transcription factor database with additional stress signals, stress-responsive transcription factor binding sites and stress-responsive genes in *Arabidopsis* and rice. Plant Cell Physiol. 54, e8. https://doi.org/10.1093/pcp/pcs185.

Nguyen, L.-T., Schmidt, H.A., von Haeseler, A., Minh, B.Q., 2015. IQ-TREE: a fast and effective stochastic algorithm for estimating maximum-likelihood phylogenies. Mol. Biol. Evol. 32, 268–274. https://doi.org/10.1093/molbev/msu300.

Rambaut, A., Drummond, A.J., Xie, D., et al., 2018. Posterior summarization in bayesian phylogenetics using Tracer 1.7. Syst. Biol. 67, 901–904. https://doi.org/10.1093/sysbio/syy032.

Roach, M.J., Schmidt, S.A., Borneman, A.R., 2018. Purge Haplotigs: allelic contig reassignment for third-gen diploid genome assemblies. BMC Bioinformatics 19, 460. https://doi.org/10.1186/s12859-018-2485-7.

Servant, N., Varoquaux, N., Lajoie, B.R., et al., 2015. HiC-Pro: an optimized and flexible pipeline for Hi-C data processing. Genome Biol. 16, 259. https://doi.org/10.1186/s13059-015-0831-x.

Simão, F.A., Waterhouse, R.M., Ioannidis, P., et al., 2015. BUSCO: assessing genome assembly and annotation completeness with single-copy orthologs. Bioinformatics 31, 3210–3212. https://doi.org/10.1093/bioinformatics/btv351.

Smid, M., Coebergh van den Braak, R.R.J., van de Werken, H.J.G., et al., 2018. Gene length corrected trimmed mean of M-values (GeTMM) processing of RNA-seq data performs similarly in intersample analyses while improving intrasample comparisons. BMC Bioinformatics 19, 236. https://doi.org/10.1186/s12859-018-2246-7.

Stanke, M., Morgenstern, B., 2005. AUGUSTUS: a web server for gene prediction in eukaryotes that allows user-defined constraints. Nucleic Acids Res. 33, W465–W467. https://doi.org/10.1093/nar/gki458.

Sun, P., Jiao, B., Yang, Y., et al., 2022. WGDI: A user-friendly toolkit for evolutionary analyses of whole-genome duplications and ancestral karyotypes. Mol. Plant 15, 1841–1851. https://doi.org/10.1016/j.molp.2022.10.018.

Sun, P., Lu, Z., Wang, Z., et al., 2024. Subgenome-aware analyses reveal the genomic consequences of ancient allopolyploid hybridizations throughout the cotton family. Proc. Natl. Acad. Sci. U.S.A. 121, e2313921121. https://doi.org/10.1073/pnas.2313921121.

Suyama, M., Torrents, D., Bork, P., 2006. PAL2NAL: robust conversion of protein sequence alignments into the corresponding codon alignments. Nucleic Acids Res. 34, W609-612. https://doi.org/10.1093/nar/gkl315.

Szukala, A., Lovegrove-Walsh, J., Luqman, H., et al., 2023. Polygenic routes lead to parallel altitudinal adaptation in *Heliosperma pusillum* (Caryophyllaceae). Mol. Ecol. 32, 1832–1847. https://doi.org/10.1111/mec.16393.

Tarailo-Graovac, M., Chen, N., 2009. Using RepeatMasker to identify repetitive elements in genomic sequences. Curr. Protoc. Bioinforma. Chapter 4, 4.10.1-4.10.14. https://doi.org/10.1002/0471250953.bi0410s25.

Vurture, G.W., Sedlazeck, F.J., Nattestad, M., et al., 2017. GenomeScope: fast reference-free genome profiling from short reads. Bioinformatics 33, 2202–2204. https://doi.org/10.1093/bioinformatics/btx153.

Wang, Z., Li, Y., Sun, P., et al., 2022. A high-quality *Buxus austro-yunnanensis* (Buxales) genome provides new insights into karyotype evolution in early eudicots. BMC Biol. 20, 216. https://doi.org/10.1186/s12915-022-01420-1.

Xie, J., Chen, Y., Cai, G., et al., 2023. Tree visualization by one table (tvBOT): a web application for visualizing, modifying and annotating phylogenetic trees. Nucleic Acids Res. 51, W587–W592. https://doi.org/10.1093/nar/gkad359.

Xu, Z., Wang, H., 2007. LTR_FINDER: an efficient tool for the prediction of full-length LTR retrotransposons. Nucleic Acids Res. 35, W265–W268. https://doi.org/10.1093/nar/gkm286.

Yang, Z., 2007. PAML 4: Phylogenetic analysis by maximum likelihood. Mol. Biol. Evol. 24, 1586–1591. https://doi.org/10.1093/molbev/msm088.

Zeng, X., Yi, Z., Zhang, X., et al., 2024. Chromosome-level scaffolding of haplotype-resolved assemblies using Hi-C data without reference genomes. Nat. Plants 10, 1184–1200. https://doi.org/10.1038/s41477-024-01755-3.

Zhang, C., Rabiee, M., Sayyari, E., et al., 2018. ASTRAL-III: polynomial time species tree reconstruction from partially resolved gene trees. BMC Bioinformatics 19, 153. https://doi.org/10.1186/s12859-018-2129-y.

Zhang, C., Zhang, J., Yang, B., et al., 2025. Chromosome-level genome assembly and annotation of *Gypsophila vaccaria*. Sci. Data 12, 818. https://doi.org/10.1038/s41597-025-05121-6.

Zheng, Y., Jiao, C., Sun, H., et al., 2016. iTAK: a program for genome-wide prediction and classification of plant transcription factors, transcriptional regulators, and protein kinases. Mol. Plant 9, 1667–1670. https://doi.org/10.1016/j.molp.2016.09.014.

Zimin, A.V., Puiu, D., Pertea, M., et al., 2025. Efficient evidence-based genome annotation with EviAnn. https://doi.org/10.1101/2025.05.07.652745.
